# Ribosomal mistranslation leads to silencing of the unfolded protein response and increased mitochondrial biogenesis

**DOI:** 10.1038/s42003-019-0626-9

**Published:** 2019-10-17

**Authors:** Dmitri Shcherbakov, Youjin Teo, Heithem Boukari, Adrian Cortes-Sanchon, Matilde Mantovani, Ivan Osinnii, James Moore, Reda Juskeviciene, Margarita Brilkova, Stefan Duscha, Harshitha Santhosh Kumar, Endre Laczko, Hubert Rehrauer, Eric Westhof, Rashid Akbergenov, Erik C. Böttger

**Affiliations:** 1 Institut für Medizinische Mikrobiologie, Universität Zürich, 8006 Zurich, Switzerland; 2 Functional Genomics Center Zurich, ETH Zürich und Universität Zürich, 8057 Zurich, Switzerland; 3 Institut de Biologie Moléculaire et Cellulaire du CNRS, Université de Strasbourg, 67084 Strasbourg, France

**Keywords:** Protein folding, Transcriptomics, Protein transport

## Abstract

Translation fidelity is the limiting factor in the accuracy of gene expression. With an estimated frequency of 10^−4^, errors in mRNA decoding occur in a mostly stochastic manner. Little is known about the response of higher eukaryotes to chronic loss of ribosomal accuracy as per an increase in the random error rate of mRNA decoding. Here, we present a global and comprehensive picture of the cellular changes in response to translational accuracy in mammalian ribosomes impaired by genetic manipulation. In addition to affecting established protein quality control pathways, such as elevated transcript levels for cytosolic chaperones, activation of the ubiquitin-proteasome system, and translational slowdown, ribosomal mistranslation led to unexpected responses. In particular, we observed increased mitochondrial biogenesis associated with import of misfolded proteins into the mitochondria and silencing of the unfolded protein response in the endoplasmic reticulum.

## Abstract (web summary)

Shcherbakov et al. investigate the consequences of ribosome mistranslation in human cells, by expressing a mutant version of the small ribosomal subunit protein RPS2. They observe activation of protein quality control pathways, but also increased mitochondrial biogenesis and silencing of the unfolded protein response in the endoplasmic reticulum.

## Introduction

The accuracy of gene expression is central to the ability of living organisms to efficiently translate genomic information into functional proteins. Errors in DNA replication occur in a frequency of ~10^–8^, and the error rate in mRNA transcription is in the rage of ~10^–6^ ^1^. By comparison, the average error rate in mRNA decoding by the ribosome has been estimated to be in the order of 10^–4^, making it the limiting factor in the accuracy of gene expression^2,3^.

Errors in mRNA decoding by prokaryotic or eukaryotic ribosomes mostly result in missense substitutions as per incorporation of mismatched tRNAs^4^. Due to the physical constraints of the mRNA-tRNA interaction, accommodation of mismatched tRNAs is limited to near-cognate tRNAs, leading mostly to missense amino acid substitutions that are conservative in nature because of the way the genetic code is arranged^5,6^. Missense errors are in part codon-dependent and affected by the levels of tRNA aminoacylation^3,7,8^.

Classical ribosomal ambiguity mutations (*ram*) in bacteria or yeast are generalized misreaders resulting in increased missense, read-through, and nonsense errors^4^. Mechanistically, *ram* mutations increase the physiological error rate of translation in a random and stochastic manner by affecting the initial phase of tRNA selection resulting in reduced discrimination against near-cognate tRNAs^9,10^. In higher eukaryotes reported defects in translational accuracy have mostly been linked to mutations which affect ribosomal accuracy in a non-random manner, i.e., mutations in specific aminoacyl-tRNA synthetases. Typically, these mutations come along with severe disease pathologies^11–15^.

Protein misfolding is a common outcome of ribosomal mistranslation and cells have developed multiple means to monitor and remove mistranslated proteins in a process collectively known as proteostasis, an interconnected network comprising more than 1000 known components in the mammalian system. Under normal conditions, molecular chaperones aid and monitor protein folding in a spatial and timely manner^16^. When the balance of protein homeostasis is disrupted, transcriptional programs dedicated to specific cellular compartments such as the cytosolic stress response and the unfolded protein response (UPR) pathways are activated in addition to the ubiquitin-proteasome pathway and autophagy to aid refolding of misfolded proteins and to remove terminally misfolded and aggregated proteins^17–19^.

Increases in the random error rate of protein synthesis have rarely been studied in higher eukaryotes. To fill this gap, we wished to establish a corresponding genetic model and to study the cellular responses by profiling the global transcriptomic and metabolomic changes together with experimental validation and functional studies. We show that mistranslation results in a proteostatic response that attenuated cytosolic protein synthesis and the cell cycle, together with increased expression of cytosolic chaperones and activation of the ubiquitin-proteasome system. In addition, ribosomal mistranslation limits protein import into the endoplasmic reticulum and silences the unfoled protein response (UPR^ER^) to circumvent UPR^ER^-triggered apoptotic pathways, and leads to increased mitochondrial biogenesis associated with import of misfolded proteins into the mitochondria.

## Results

### Identification of a *ram* mutation in higher eukaryotes

Mutation *RPS2* S200Y in the lower eukaryote *Saccharomyces cerevisiae* (uS5) is a well-known ribosomal ambiguity mutation (*ram*) that disrupts the RPS2*(*uS5*)*-RPS9 *(*uS4*)* interface on the small ribosomal subunit (Supplementary Fig. 1)^20–22^. By aligning the *RPS2* (uS5) sequences of *S. cerevisiae* and *Homo sapiens*, we identified A226Y as the homologous human *RPS2* mutation, corresponding to *RPS2*-S200Y in yeast. Structural modeling of the human RPS2*(*uS5*)*-RPS9*(*uS4*)* interface suggested that the *RPS2*-A226Y mutation would result in steric hindrance between the tyrosine residue and the α-helix in the protein C-terminal domain, affecting the conformation of RPS2 and thus its interaction with RPS9 (Supplementary Fig. 1e, f). Based on the modeling results, we hypothesized that *RPS2* A226Y would confer mistranslation in higher eukaryotes.

To assess whether the identified mutation confers mistranslation in human ribosomes, we generated stable transfected HEK293 cells constitutively expressing wild-type *RPS2* or mutant *RPS2-*A226Y resulting in cells termed wild-type (*cells transfected with RPS2-wt*) and A226Y (*cells transfected with RPS2-A226Y*), respectively, untransfected HEK293 cells were termed HEK293 cells; this nomenclature is used throughout the paper. Transfection with myc-tagged wild-type *RPS2* and mutant A226Y *RPS2* genes showed that the myc-tagged RPS2 was enriched in the ribosomal fraction, indicating that the transgenic RPS2 protein was being incorporated into functional ribosomes. Additional experiments demonstrated that the transfected RPS2 protein is incorporated into actively translating polysomes (Supplementary Fig. 2a, b).

**Fig. 1:**
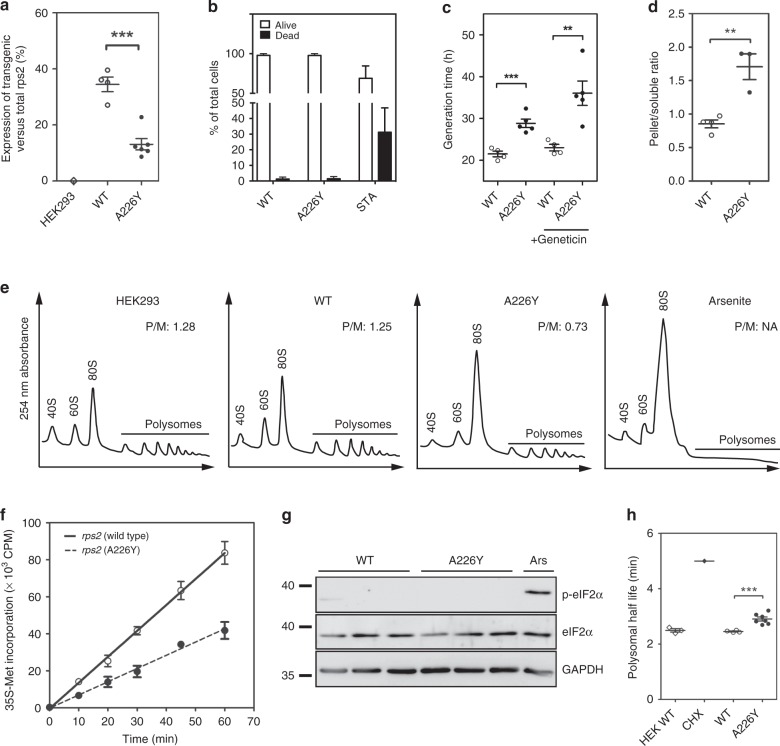
Characterization of *RPS2* A226Y mutant cells. **a** *RPS2* transgene expression as a fraction of total *RPS2* mRNA expression using TaqMan qRT-PCR (*N* ≥ 4). HEK293 = untransfected HEK cells; WT = HEK cells transfected with wt *RPS2*; A226Y = HEK cells transfected with *RPS2* A226Y. **b** Trypan blue exclusion assay for the determination of cell viability (*N* = 6). Staurosporine (STA, 10 μM, 24 h) used as positive control for cell death. **c** Generation time with and without geneticin treatment as determined by Alamar blue assay (4 ≤ *N* ≤ 6). **d** Firefly luciferase aggregation assay (*N* ≥ 3). A representative western blot can be found in Supplementary Fig. 2d. **e** Polysome profiling of HEK293 (non-transfected), *RPS2* WT, *RPS2* A226Y, and 0.5 mM arsenite-treated HEK WT cells. The polysome to monosome (P/M) ratios were calculated using the area under the curve of the polysome and the 80 S monosome peaks. Representative figures are shown (*N* = 3). **f** In vivo protein synthesis determined by radiolabelled methionine (^35^S-Met) incorporation (*N* = 3). **g** Western blot to determine the phosphorylation status of eIF2α. Antibodies against phosphorylated and unphosphorylated eIF2α were used. Arsenite (Ars, 0.5 mM, 1 h) used as positive control, GAPDH as loading control (*N* = 3). **h** Polysome half-life as derived from the polysome runoff assay (*N* ≥ 4). Treatment with elongation inhibitor cycloheximide (CHX) used as positive control. See also Supplementary Fig. 2e-g. **p < 0.01, ***p < 0.005. *N* = number of independent clones analyzed for each genotype; for each clone 3 technical replicates were done. Mean ± SEM is given

To assess the expression levels of transgenic versus endogenous *RPS2*, we performed Taqman qRT-PCR. mRNA levels for transfected *RPS2*-wt ranged between 30–40% of total *RPS2* expression. In contrast, *RPS2*-A226Y transgenic mRNA was expressed at only ~10% of total *RPS2* expression (Fig. 1a). The reduced expression of the *RPS2*-A226Y transgene was mirrored by expression levels of an IRES-GFP-reporter fused downstream of transgenic *RPS2* as an alternative method for transgene quantification (Supplementary Fig. 2c).

The ability of *RPS2* mutation A226Y to induce misreading and stop codon read-through was assessed using the dual-luciferase mistranslation reporter system as previously described^23,24^. In this assay, the active site H245 of firefly luciferase (Fluc) was mutated from CAC to near-cognate CGC and to non-cognate AGA, resulting in a non-functional protein. To measure read-through, stop codon TGA was introduced at D357 of Fluc to produce a truncated and non-active Fluc. The misreading agent geneticin was included as a positive control for both assays. In comparison to *RPS2* wt, we found that *RPS2*-A226Y mutant cells showed increased levels of near-cognate misreading (2.2-fold) and stop codon read-through (5.4-fold) (Table 1). The finding of *RPS2*-A226Y-mediated ribosomal misreading provides further evidence that transfected *RPS2* is incorporated into ribosomes and present in actively translating ribosomes. As previously reported for *ram* mutations in *E. coli* and yeast, *RPS2* mutation A226Y only affected misreading of near-cognate but not of non-cognate codons (AGA column, Table 1). Further, A226Y-mediated read-through was found to extend to disease-associated nonsense codons in the context of Hurler disease and cystic fibrosis (Table 1).

**Table 1:** Misreading and read-through, Hurler disease, and cystic fibrosis associated read-through

	Misreading		Read-through
Transfectants	CGC^a^	AGA^b^	UGA
HEK untreated	100	100	100
Geneticin [400 µM]	**562** **±** **59**	104 ± 21	**1963** **±** **126**
RPS2 WT	112 ± 8	114 ± 7	109 ± 5
RPS2 A226Y	**246** **±** **15**	111 ± 7	**593** **±** **35**

	Read-through		
	UAG	UAG	UAG
Hurler disease context	Control	W392X	W402X
RPS2 WT	123 ± 12	156 ± 12	131 ± 31
RPS2 A226Y	**420** **±** **82**	**628** **±** **35**	**711** **±** **142**
Gentamicin [500 µM]	**489** **±** **53**	**285** **±** **16**	**307** **±** **24**
Paromomycin [4 mM]	**901** **±** **138**	**708** **±** **51**	**759** **±** **203**

	UGA	UGA	UGA
Cystic fibrosis context	Control	G542X	W1282X
RPS2 WT	119 ± 16	109 ± 5	119 ± 7
RPS2 A226Y	**419** **±** **89**	**206** **±** **16**	**322** **±** **31**
Gentamicin [500 µM]	**244** **±** **28**	**217** **±** **18**	**173** **±** **14**
Paromomycin [4 mM]	**376** **±** **64**	**203** **±** **12**	**285** **±** **48**

Values are shown as % to HEK WT untreated. Bold text indicates values significantly different from the WT control (*p* < 0.05). Misreading agents geneticin, gentamicin, and paromomycin used as positive controls

^a^near-cognate codon (His CAC → Arg CGC)

^b^non-cognate codon (His CAC → Arg AGA)

Together, these data identify the replacement of valine by tyrosine at RPS2 aa position 226 as a *ram* mutation and demonstrate that the transfected RPS2 is incorporated into actively translating ribosomes.

### Effects of *RPS2* A226Y on translation

Cell viability was not measurably affected in the *RPS2*-A226Y mutants (Fig. 1b), however, we detected a significantly longer generation time compared to *RPS2* wt transfected cells (Fig. 1c). This loss of fitness, a hallmark of *ram* mutations, was exacerbated by the aminoglycoside geneticin (Fig. 1c).

To assess whether A226Y-induced mistranslation resulted in increased protein aggregation in the cell, we used cells transfected with a misfolding-prone firefly luciferase reporter^25^. Using antibodies against Fluc, we performed western blots of the soluble and pellet fractions of cellular extracts. The distribution of Fluc shifted towards the insoluble pellet fraction for the A226Y mutants compared to *RPS2* wt indicating that the misreading mutation A226Y induced higher levels of luciferase aggregation (Fig. 1d, Supplementary Fig. 2d).

Translational activity in the A226Y mutants was substantially reduced as assessed by in vivo ^35^S-methionine incorporation (Fig. 1f). Polysome profiling demonstrated that the decrease in protein synthesis was accompanied by an increase in monosomes in the A226Y mutants (Fig. 1e). To investigate how translational activity was regulated, we first studied eukaryotic translation initiation factor 2α (eIF2α) phosphorylation as an indicator of initiation inhibition^26^. In contrast to arsenite, a known inducer of eIF2α phosphorylation, western blotting failed to detect differences in eIF2α phosphorylation between A226Y and wild-type cells suggesting that translation was not regulated at the level of initiation (Fig. 1g).

Next, we used polysome run-off assays to study translation elongation. The translation inhibitor harringtonine stalls initiating ribosomes at the start codon while allowing initiated ribosomes to continue elongating and run off their transcripts. The time it takes for polysome depletion after harringtonine treatment correlates with translation elongation speed^27^ (Supplementary Fig. 2e). Compared to *RPS2* wt, A226Y mutants showed slower polysome runoff, i.e., a reduced elongation rate (Fig. 1h, Supplementary Fig. 2f). Cycloheximide, a known inhibitor of translation elongation was included as a positive control for elongation slowdown (Supplementary Fig. 2g).

### Transcriptome profiling of A226Y-induced mistranslation

To examine globally how the mammalian cell responds to chronic mistranslation, we analyzed the transcriptome response of A226Y mutants using RNA sequencing (RNA-Seq). 5550 genes were differentially regulated in the A226Y mutants compared to *RPS2* wt (*p* < 0.05) (Supplementary Fig. 3a, b). Process network and Gene Ontology (GO) enrichment analyses identified significantly regulated processes involved in proteolysis, protein folding, translation, apoptosis, cell cycle regulation and DNA damage repair (Supplementary Fig. 3c, d). As expected, mistranslation results in activation of various protein quality control pathways, as revealed, for example, by elevated transcript levels for cytosolic chaperones and the ubiquitin-proteasome system. However, two findings were unanticipated. First, transcripts for the UPR were decreased in the A226Y mutants. Second, pathways involved in mitochondrial translation and protein import were significantly increased in the A226Y mutants (Supplementary Fig. 3d).

### Translation and protein quality control pathways

We found decreased transcript levels of genes involved in cytosolic protein synthesis, including structural ribosomal proteins, aminoacyl tRNA synthetases, and elongation factors (Supplementary Fig. 4a, b), consistent with our experimental data showing reduced translational activity and elongation slowdown. We also found increased transcript levels of ribosomal release factors eRF1 and eRF3 (Supplementary Fig. 4b), most likely reflecting a response to A226Y-mediated increased stop codon read-through.

In addition, we observed elevated transcript levels for cytosolic chaperones and proteasome components (Supplementary Fig. 4c, d). Thus, A226Y-induced mistranslation upregulates cytosolic protein quality control pathways. We corroborated these findings by western blot analysis to detect ubiquitination at the protein level and by measuring the proteolytic activity of the 26S proteasome. The A226Y mutants showed significantly increased levels of ubiquitination and of proteasomal degradation compared to the wild-type (Supplementary Fig. 4e–g). In line with these observations we found that A226Y-mediated mistranslation increases susceptibility to proteasomal inhibitors. Treatment with proteasome inhibitor MG132 resulted in significantly increased cell death in the A226Y mutants as compared to *RPS2* wt (Supplementary Fig. 4h).

KEGG analysis revealed reduced gene transcript levels for pathways involved in biosynthesis and metabolism of amino acids (Supplementary Tables 1, 2). In combination with increased transcript levels of genes involved in proteasome-mediated proteolysis, this observation suggests an extensive recycling of amino acids for protein synthesis. To complement our gene expression data, we assessed the metabolic changes in the A226Y mutants. Metabolome analysis identified significantly increased levels of dipeptides (remnants of proteolytic activity), while metabolites involved in amino acid metabolism and gamma-glutamate-dependent amino acid import were significantly reduced (Supplementary Fig. 4i, j, Supplementary Tables 1, 2, 3). These findings corroborate the transcriptome data suggesting activation of proteasome-mediated proteolysis and increased recycling of amino acids.

### Chronic mistranslation results in G1/S transition defect

**Fig. 2:**
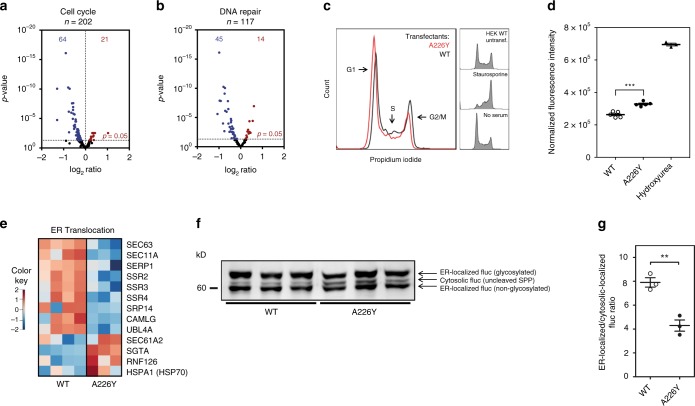
Regulation of DNA repair, cell cycle and ER transport in the A226Y mutants. **a**, **b** Volcano plots showing genes involved in cell cycle regulation and DNA damage repair. Blue dots represent significantly downregulated genes, red dots represent significantly upregulated genes (*N* ≥ 3, *p* < 0.05). **c** Cell cycle analysis using propidium iodide (50 μg/ml) staining. Representative profiles of A226Y versus WT are shown (*N* = 6). Cells were incubated (24 h) in serum-free medium as a positive control for G1 arrest, staurosporine (STA, 200 nM, 24 h) used as G2 arrest control. **d** Levels of CDK2 protein phosphorylated TYR15 (CDK2 pTYR15) as determined by ELISA. CDK2 pTYR15 is elevated in G1/S phase. 1 mM hydroxyurea used as positive control for G1/S arrest (*N* = 5). **e** Heatmap of the significantly regulated genes (*p* < 0.05) involved in ER translocation. **f** Representative western blot of the ER-firefly luciferase (prolactin signal sequence used as target signal for ER). Three different bands are identified: cytosolic Fluc (uncleaved SRP), ER-localized Fluc (non-glycosylated), ER-localized Fluc (glycosylated). **g** Ratio of ER-localized/cytosolic localized Fluc as derived from quantification of western blot analysis (*N* = 3). The full list of cell cycle, DNA repair, ER transport and PQC genes were compiled from available literature and can be found in Supplementary Data 1. ***p < 0.001. **a**, **b**, **e** For transcriptome analysis 4 independent clones of WT-transfected cells and 3 independent clones of A226Y-transfected cells were used; (**c**, **d**, **f**, **g**) *N* = number of independent clones analyzed for each genotype; for each clone 3 technical replicates were done. Mean ± SEM is given

Process network enrichment analysis revealed an enrichment of cell cycle networks in the A226Y mutant. In short, we found increased levels of CDK inhibitors together with decreased transcript levels for genes involved in mitosis, DNA replication and repair, all suggestive of a G1/S transition defect in the mutant (Fig. 2a, b). Compared to the control, A226Y mutants showed a higher proportion of cells in G1/S (Fig. 2c) when DNA was stained with propidium iodide. This observation was corroborated by the higher amounts of Cdk2-pTyr15 protein (G1/S enriched protein) detected in the A226Y mutants compared to wild-type cells^28^ (Fig. 2d).

### Chronic mistranslation reduces protein import into ER

Another finding from our GO enrichment analysis was the decreased transcript levels for the ER protein targeting machinery (Supplementary Fig. 3d). Protein transport into the ER happens via three routes: the signal recognition particle-dependent (SRP) pathway, the SRP-independent pathway, and guided entry of tail-anchored protein (GET)^29,30^. Global transcriptome profiling suggested that the components directly involved in translocation across the ER membrane for all three routes were reduced in the A226Y mutant. This is indicated by significantly reduced transcript levels of genes encoding for the main players involved: SERP1, SPase, SSRs, SEC63, and CAMLG, with the marked exception of SEC61, possibly due to its suggested role in ER dislocation, i.e., the export of misfolded proteins back to the cytosol for proteolytic degradation^31^ (Fig. 2e). To challenge this finding, we used a Fluc reporter carrying a prolactin ER targeting sequence and a KDEL ER retention sequence to detect differences in ER trafficking between the A226Y mutant and *RPS2* wt. Western blotting against Fluc in the total cell lysates revealed a significantly lower ER-localized/cytosolic Fluc ratio in A226Y mutants when compared to controls, indicating that transport into the ER is reduced in A226Y mutants (Fig. 2f, g, Supplementary Fig. 5). In addition, we consistently found elevated transcript levels of *SGTA* and *RNF126*, key components of the mislocalized protein (MLP) degradation pathway^32,33^, suggesting a re-routing of ER-destined proteins to the proteasome via pre-emptive quality control pathways^34^.

### Chronic mistranslation silences the UPR

**Fig. 3:**
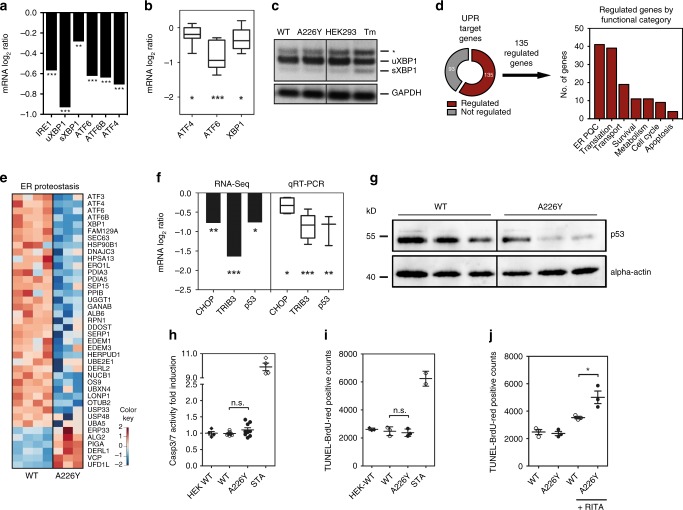
The unfolded protein response (UPR) of the A226Y mutant. **a** mRNA levels (RNA-Seq) of UPR signaling factors (*N* ≥ 3, *p* < 0.05). **b** mRNA expression levels (qRT-PCR) of selected UPR signaling factors (6 ≤ *N* ≤ 8). **c** XBP1-splicing in A226Y mutants. Tunicamycin (Tm) as positive control, GAPDH as loading control. The asterisk (*) indicates the position of a hybrid amplicon, a representative experiment is shown (*N* = 3). **d** The pie chart represents the UPR target genes activated by transcription factors ATF6, XBP1, and ATF4 and the significantly regulated fraction (135/228) in the A226Y mutants. The histogram classifies the 135 genes according to functional categories (*N* ≥ 3, *p* < 0.05). **e** Heat map representing the significantly regulated UPR target genes (*p* < 0.05) involved in ER protein quality control (ER-PQC). UPR target genes compiled from available literature and can be found in Supplementary Data 1. **f** mRNA levels of downstream UPR apoptotic factors CHOP, TRIB3, and p53 by RNA-Seq and qRT-PCR (3 ≤ *N* ≤ 5, *p* < 0.05). **g** Representative western blot of p53 levels in A226Y versus WT. Anti-p53 antibodies were used, actin as loading control. (*N* = 3). **h** Caspase 3/7 splicing activity assay. Values normalized to HEK-WT (*N* ≥ 4). **i** TUNEL-BrdU-Red assay (*N* = 3). **j** Activation of p53 by treatment with MDM2-inhibitor RITA (10 µM for 18 h). Level of apoptosis determined using the TUNEL assay (*N* = 3). *p < 0.05, **p < 0.01, ***p < 0.005. (**a**, **d**, **e**, **f**) For transcriptome analysis 4 independent clones of WT-transfected cells and 3 independent clones of A226Y-transfected cells were used; (**b**, **c**, **g**-**j**) *N* = number of independent clones analyzed for each genotype; for each clone 3 technical replicates were done. Mean ± SEM is given

Protein stress in the ER is detected by three UPR sensors (PERK, IRE1, and ATF6) which activate a transcriptional response that temporarily reduces protein synthesis while improving the folding and clearance capacity of the ER^17^. Surprisingly, we found reduced transcript levels of key UPR signaling factors, i.e., IRE1, XBP1 – a transcription factor activated by IRE1, ATF6, and ATF4, a central transcriptional regulator downstream of the PERK signaling pathway (Fig. 3a). Decreased expression of these UPR activation pathways in the A226Y mutants was confirmed using qRT-PCR (Fig. 3b). In addition, XBP1 splicing, a key step in IRE1 signaling, showed no significant difference between A226Y and the wild-type (Fig. 3c). Taken together, the data suggest that the UPR sensors responsible for transcriptionally activating the UPR were downregulated in the A226Y mutants compared to the *RPS2* wt.

Next, we examined downstream UPR effectors transcriptionally activated by PERK, IRE1, and ATF6 signaling. One hundred and thirty-five from a total of 228 reported UPR target genes were differentially regulated in the A226Y mutants compared to wild-type. Interestingly, the majority of those genes belonged to one of two categories: ER protein quality control (41/135) or translation (39/135) (Fig. 3d). In the A226Y mutants, decreased gene transcript levels were observed for all components of the ER protein quality control, including ER-resident chaperones, N-linked glycosylation-related proteins, protein disulfide isomerases (PDIs), peptidyl-prolyl isomerases (PPIs), and components of the ER-associated degradation (ERAD) pathway (Fig. 3e). To compare our findings with an acute UPR^ER^ inducer, we treated HEK293 cells with the aminoglycoside geneticin. Interestingly, both UPR effector and target genes of geneticin-treated cells were strongly upregulated, in sharp contrast to the A226Y response (Supplementary Fig 6).

### Downregulation of the UPR to avoid triggering of apoptosis

We hypothesized that UPR^ER^ is silenced during chronic ribosomal mistranslation in order to avoid triggering UPR-mediated apoptosis. Although the exact molecular mechanisms involved in UPR^ER^ triggered apoptosis are not fully elucidated, extensive ER stress has been reported to trigger apoptosis using one of two pathways. The first route is linked to transcription factor CHOP (also named GADD153)^35^. CHOP is regulated by PERK activation and activates downstream effectors like TRIB3 to trigger mitochondria-dependent apoptosis^36^. Interestingly, PERK-deficient or CHOP-deficient cells still undergo apoptosis during ER stress, mediated by p53 activation of BH3-only pro-apoptotic BIK^37^.

Based on RNA-Seq and qRT-PCR data, transcript levels of CHOP, TRIB3 and p53 were all significantly downregulated in the A226Y mutant compared to the wild-type (Fig. 3f). Western blotting of p53 similarly showed reduced levels of p53 in the A226Y mutants (Fig. 3g). Consistently, the A226Y mutants showed no significant differences in apoptosis compared to wild-type when determined by caspase 3/7 activity and TUNEL staining (Fig. 3h, i). On the other hand, geneticin-treated cells which showed activation of the three arms of the UPR^ER^ have increased levels of CHOP and readily underwent apoptosis (Supplementary Fig. 6d, e). In further support of our hypothesis, mutant A226Y cells show heightened susceptibility to p53-mediated apoptosis. Thus, treatment with p53 activator RITA induced a significantly higher level of apoptosis in the A226Y mutants compared to the wild-type (Fig. 3j). These results, taken together provide evidence that the UPR^ER^ is silenced during chronic protein mistranslation to avoid triggering apoptosis.

### Linking the mitochondria to proteostasis

**Fig. 4:**
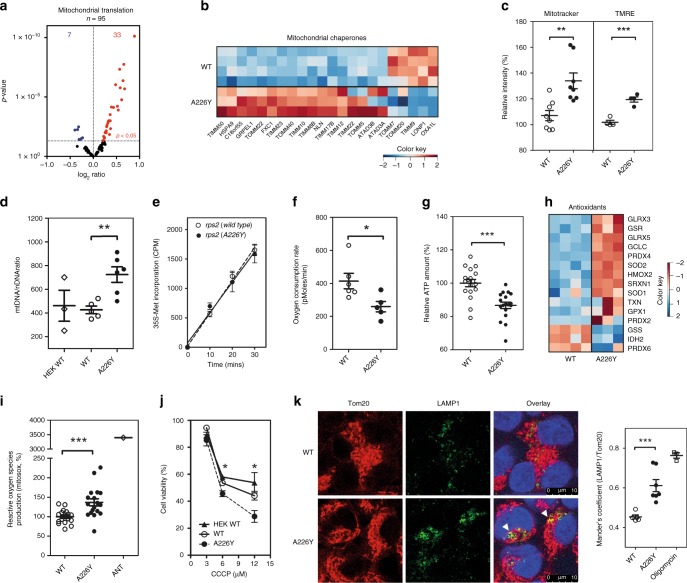
Characterization of the mitochondria in the A226Y mutant. **a** Volcano plot of mitochondrial ribosome structural genes and tRNAs synthetases (RNA-Seq) (*N* ≥ 3, *p* < 0.05). **b** Heatmap of significantly regulated mitochondrial chaperones (*p* < 0.05). **c** Mitochondrial mass measured by Mitotracker Deep Red FM and TMRE (FACS; mean fluorescence ± SEM; 6 ≤ *N* ≤ 8). **d** Mitochondrial DNA (mtDNA) to nuclear DNA (nDNA) ratio (3 ≤ *N* ≤ 6). **e** In vivo mitochondrial translation using ^35^S-Met incorporation (*N* = 3). **f** Oxygen consumption rate (OCR) measured using Seahorse XF-24. Mean ± SEM (*N* ≥ 5). **g** Relative ATP levels (4 ≤ *N* ≤ 6). **h** Heatmap of significantly regulated antioxidant genes (*p* < 0.05). **i** Detection of reactive oxygen species (ROS) production using MitoSOX staining and FACS. Antimycin (ANT, 10 μg/ml) used as positive control (*N* = 6). **j** Dose-response curve of CCCP treated cells using Alamar blue staining (N = 3). **k** Immunofluorescent images of mitochondria (TOM20, red) and lysosome (LAMP1, green). DAPI used to visualize cell nuclei (blue). Mitophagy is visualized by fusion of mitochondria with lysosomes (yellow) and are marked with white triangles. Histogram represents the quantification of LAMP1 and TOM20 using Mander’s Coefficient (*N* = 5). The full list of mitochondrial chaperones and antioxidant genes were compiled from available literature and can be found in Supplementary Data 1. *p < 0.05, **p < 0.01, ***p < 0.005. **a**, **b**, **h** For transcriptome analysis 4 independent clones of WT-transfected cells and 3 independent clones of A226Y-transfected cells were used; (**c**–**g**, **i**–**k**) *N* = number of independent clones analyzed for each genotype; for each clone 3 technical replicates were done. Mean ± SEM is given

In sharp contrast to limiting protein import into the ER, we found increased transcript levels for components of the mitochondrial import machinery, chaperones, mitochondrial organization and protein synthesis (Fig. 4a, b, Supplementary Fig. 3d, 7a). We stained cells with Mitotracker DeepRed (total mitochondrial content) and tetramethylrhodamine ethyl ester (TMRE-mitochondrial membrane potential). Increased Mitotracker DeepRed and TMRE staining were observed by FACS in the A226Y mutant, indicative of increased mitochondrial mass (Fig. 4c). We also estimated the number of mitochondria by quantifying relative amounts of mitochondrial DNA (mtDNA) and nuclear DNA (nDNA) as previously described^38^. The A226Y mutants showed a significantly higher mtDNA/nDNA ratio when compared to wild-type (Fig. 4d).

We next examined whether the A226Y mutation affected translation of mRNA derived from mtDNA. In contrast to cytoplasmic translation, in vivo mitochondrial protein synthesis was not affected by the A226Y mutation (Fig. 4e, Supplementary Fig. 7b). Despite having more mitochondria per cell, we found that basal respiration as determined by oxygen consumption rate and ATP production was significantly reduced in the A226Y mutant suggestive of impaired mitochondrial function^39^ (Fig. 4f, g). We looked for evidence of oxidative stress and found an increase in reactive oxygen species and an upregulation of the majority of antioxidant genes in the A226Y mutants (Fig. 4h, i). We also found that the A226Y mutation was associated with enhanced mitophagy, as assessed by immunofluorescence microscopy quantifying the overlap of TOM20 (mitochondria) and LAMP1 (lysosomes) staining (Fig. 4k).

While these observations are suggestive of classical mitochondrial dysfunction, a recent report has proposed an active role for mitochondria during proteostasis in yeast, where cytosolic aggregation-prone proteins become imported and degraded within the mitochondria^40^. As the import of mitochondrial proteins relies on the electrochemical potential of the inner mitochondrial membrane, we treated cells with the mitochondrial uncoupler CCCP, a disruptor of the inner mitochondrial membrane potential, to study the cellular susceptibility to pharmacological impairments of mitochondrial import^41^. Compared to *RPS2* wt cells, the A226Y mutants were significantly more susceptible to treatment with CCCP, as detected by cell viability staining using Alamar Blue (Fig. 4j). To further explore the relationship between mistranslation and mitochondrial biogenesis observed in the A226Y mutants, we studied the response to geneticin, a known misreading agent. We observed similar responses in mitochondrial import, translation, biogenesis and mitophagy as in the A226Y mutants (Supplementary Figs. 7c–e, 8a, c). In addition, an increase in the load of misfolded proteins at the post-ribosomal level induced by inhibition of proteasome function, led to an increase in mitochondrial mass and mitophagy (Supplementary Fig. 8b, c). Altogether, these results connect mistranslation with increased mitochondrial biogenesis and mitophagy, thus providing circumstantial evidence for a mitochondrial role in proteostasis.

### Mitochondrial localization of misfolded cytosolic proteins

**Fig. 5:**
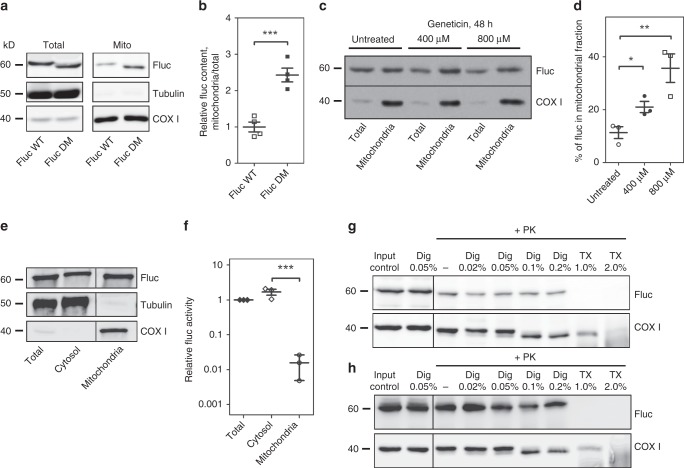
Misfolded proteins localize to mitochondria. Cell fractionations of HEK293 cells transfected with misfolded-prone FlucWT and mutant aggregation-prone FlucDM. **a** Immunoblots and (**b**) quantification showing FlucWT and FlucDM content in the isolated mitochondrial fraction in comparison to total cellular content (*N* = 4). Anti-Fluc antibody was used to visualize luciferase protein; tubulin and COX1 antibodies were used as controls. Samples were loaded to provide approximately equal amounts of COX1 for comparison. **c**, **d** Treatment with geneticin increases the proportion of Fluc recovered in the mitochondrial fraction. **c** Representative western blot of HEK293 cells expressing cytosolic FlucWT and treatment with geneticin (400 µM and 800 µM). **d** Quantification of Fluc recovered in the mitochondrial fraction. The amount of Fluc in the mitochondria fraction was quantified in relation to the amount of Fluc in the total fraction, and normalized to COX1: $\left( {\frac{{{\mathrm{Fluc}}\,{\mathrm{mitochondria}}}}{{{\mathrm{COXI}}\,{\mathrm{mitochondria}}}}:\frac{{{\mathrm{Fluc}}\,{\mathrm{total}}}}{{{\mathrm{COXI}}\,{\mathrm{total}}}}} \right) \times {\mathrm{100}}$ (*N* = 3). **e**, **f** Measurements of specific luciferase activity. **e** Western blot of HEK293 cells expressing cytosolic FlucWT to demonstrate the distribution of the Fluc in the different cellular fractions. Samples were loaded to provide approximately equal amounts of firefly luciferase for comparison. **f** Firefly luciferase enzymatic activity in the different fractions given in panel **e**; the specific luciferase activity was obtained by relating the enzymatic activity of each fraction to the corresponding band intensity quantification by western blot. The specific activity of total fraction was set as 1, the specific activity of the mitochondrial fraction is 0.016 ± 0.011; *N* = 3. **g** Representative western blot of mitochondrial fractions isolated from HEK293 cells expressing cytosolic FlucWT treated with proteinase K alone or in combination with digitonin or Triton X-100. **h** Representative western blot of mitochondrial fractions isolated from HEK293 cells expressing mitochondria targeted MTS-FlucWT treated with proteinase K alone or in combination with digitonin or Triton X-100. *p < 0.05, **p < 0.01. *N* = number of independent transient transfections for each vector; for each transfection 3 technical replicates were done. Mean ± SEM is given

To examine whether mistranslated proteins associate with mitochondria we performed fractionation experiments of cells transfected with misfolding-prone wild-type luciferase (WT-Fluc) and a mutant aggregation-prone luciferase derivative (DM-Fluc)^40^. Purification of mitochondria and western blotting demonstrated physical association of misfolded-prone WT-Fluc with mitochondria, association with the mitochondrial fraction was more pronounced for the mutant DM-Fluc (Fig. 5a, b). Treatment with the misreading agent geneticin resulted in increased localization of WT-Fluc to mitochondria (Fig. 5c, d). Functional assays of specific enzymatic luciferase activity demonstrated that the luciferase protein in the mitochondrial fraction shows drastically reduced enzymatic activity as compared to the cytosolic luciferase, indicating that the vast majority of mitochondrially localized luciferase is misfolded and enzymatically inactive (Fig. 5e, f).

To examine the association of misfolded Fluc with mitochondria we treated purified mitochondrial fractions from cells expressing WT-Fluc with proteinase K to eliminate misfolded Fluc attached to the outside of mitochondria (Fig. 5g). The mitochondrial outer and inner membranes were permeabilized using digitonin and Triton X-100, respectively^42^. Western blot analysis showed that substantial amounts of Fluc stayed intact after treatment with proteinase K alone or in combination with digitonin permeabilizing the outer mitochondrial membrane, and is degraded only after treatment with proteinase K in combination with Triton X-100, dissolving both outer and inner membrane. COX1 was used as a control for a protein localized in the mitochondrial matrix. As a further control we used WT-Fluc with a mitochondrial targeting signal (MTS-WT-Fluc). The mitochondrial targeting signal directs the import of the Fluc reporter into the mitochondria. MTS-WT-Fluc in mitochondrial fractions was protected from treatment with proteinase K alone or in combination with digitonin and was degraded only upon a combined treatment with proteinase K and Triton X-100 (Fig. 5h).

The results from the cell fractionation experiments indicate that misfolded luciferase associates with and becomes imported into mitochondria. We performed additional experiments to visualize localization of misfolded luciferase and to substantiate this conclusion more directly. We employed the split GFP system, where the first ten β-strands of GFP (GFP_1–10_), fused with mCherry, are targeted to mitochondria through linkage with a mitochondria targeting sequence (MTS-mCherry-GFP_1–10_), while the eleventh β-strand (GFP_11_) is linked to a reporter. Mitochondrial GFP fluorescence is expected only if the GFP_11_-linked reporter enters mitochondria. As reporter we used glutathione S-transferase (GST), as control for a stable cytosolic protein, the misfolded-prone wild-type luciferase (WT-Fluc), the mutant aggregation-prone DM-Fluc, and MTS-GFP_11_ as positive control. Each construct was cotransfected with a plasmid expressing MTS-mCherry-GFP_1–10_. For further localization of the split-GFP signal and to exclude aberrant expression of MTS-mCherry-GFP_1–10_ we used staining with TOM20, a mitochondrial protein of the outer membrane.

**Fig. 6:**
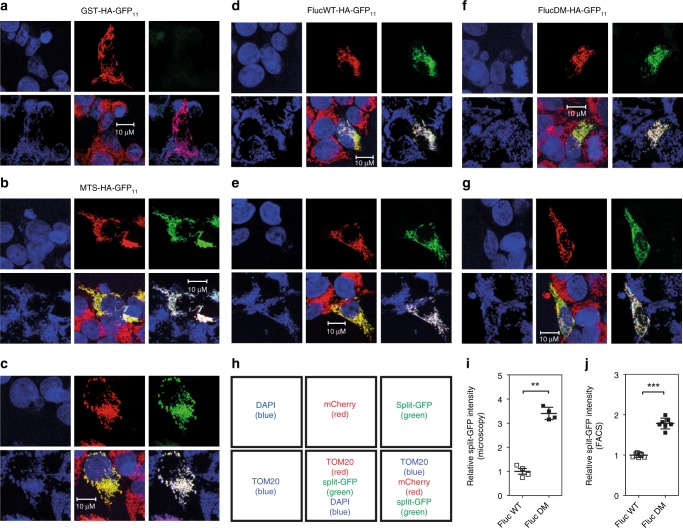
Association of misfolded proteins with mitochondria. **a**–**g** Images of transfected HEK293 cells–top 3 panels: DAPI (blue), mCherry (red), split-GFP (green); bottom 3 panels: mitochondria (TOM20 antibody staining, blue), overlay (DAPI blue, TOM20 red, split-GFP green), overlay (TOM20 blue, mCherry red, split-GFP green); see also scheme in panel **h**. Cotransfection with GST-HA-GFP_11_/MTS-mCherry-GFP_1–10_ was used as negative control (**a**), cotransfection with MTS-HA-GFP_11_/MTS-mCherry-GFP_1–10_ was used as positive control (**b**, **c**). **d**–**g** Representative images of HEK293 cells cotransfected with misfolded-prone FlucWT-HA-GFP_11_/MTS-mCherry-GFP_1–10_ (**d**, **e**) and mutant aggregation-prone FlucDM-HA-GFP_11_/MTS-mCherry-GFP_1–10_ (**f**, **g**). **i** Quantification of normalized GFP/mCherry ratio assessed by wide-field fluorescent microscopy, median fluorescence ± SEM (*N* = 4). **j** Quantification of split-GFP signal by FACS. Normalized split-GFP/mCherry ratio relative to Fluc expression (median fluorescence ± SEM; *N* ≥ 6). **p < 0.01, ***p < 0.005. *N* = number of independent transient transfections for each vector; for each transfection 3 technical replicates were done

**Fig. 7:**
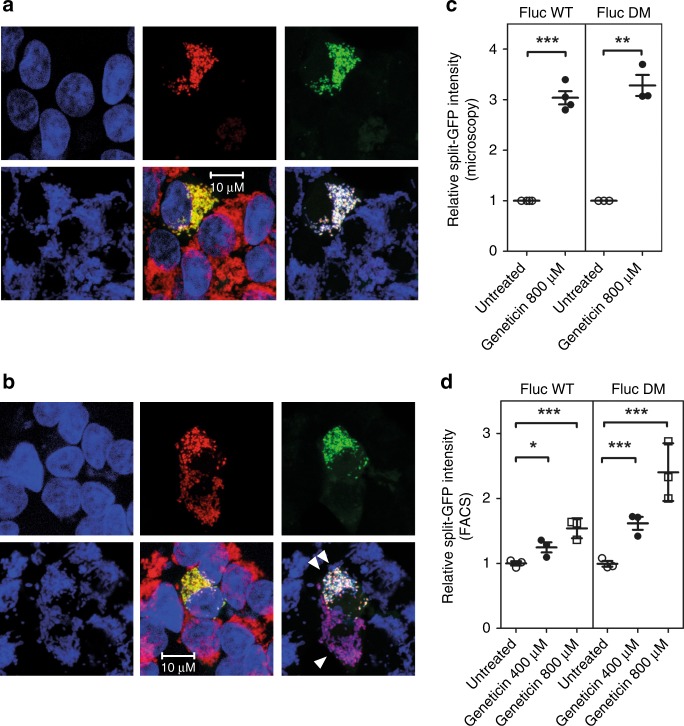
Mistranslation increases the proportion of misfolded Fluc associated with mitochondria. HEK293 cells cotransfected with FlucWT-HA-GFP_11_/MTS-mCherry-GFP_1–10_ and FlucDM-HA-GFP_11_/MTS-mCherry-GFP_1–10_ were treated with the misreading agent geneticin–images are arranged as in Fig. 6. **a**, **b** Representative images of cells transfected with FlucWT-HA-GFP_11_ and treated with 800 µM geneticin for 24 h. Panels are arranged as in Fig. 6a–g. Cell expressing MTS-mCherry-GFP_1–10_ is marked by a single white triangle, cell co-expressing both FlucWT-HA-GFP_11_ and MTS-mCherry-GFP_1–10_ is marked by two white triangles. **c** Quantification of normalized GFP/mCherry ratio as assessed by wide-field fluorescence microscopy, median fluorescence ± SEM (*N* ≥ 3). **d** Treatment with 400 µM and 800 µM increases association of Fluc with mitochondria in a dose-dependent manner; quantification of normalized split-GFP/mCherry ratio relative to Fluc expression by FACS (median fluorescence ± SEM; *N* = 6). *p < 0.05, **p < 0.01, ***p < 0.005. *N* = number of independent transient transfections for each vector; for each transfection 3 technical replicates were done

The positive control, MTS-GFP_11_, showed a prominent mitochondrial GFP signal, whereas the negative control, GST-GFP_11_, showed no mitochondrial split-GFP signal (Fig. 6a–c). Misfolding-prone WT-Fluc-GFP_11_ and mutant DM-Fluc-GFP_11_ showed an increasingly strong GFP signal, which colocalized with TOM20, demonstrating that misfolding-prone and aggregation-prone cytosolic reporter proteins associate with mitochondria (Fig. 6d–j). We next treated HEK293 cells expressing WT-Fluc-GFP_11_ or DM-Fluc-GFP_11_ with the misreading agent geneticin to examine whether drug-induced mistranslation affects mitochondrial localization of the Fluc-GFP_11_ reporters. Treatment with geneticin resulted in significantly increased split GFP fluorescence for both WT-Fluc and DM-Fluc as assessed by fluorescence microscopy and FACS analysis (Fig. 7).

## Discussion

We know surprisingly little about the cellular response to changes in the average error rate of mRNA decoding, and how the various individual pathways interact to maintain cell function when ribosomal accuracy is impaired. Towards this end, we generated what is to our knowledge, the first ever reported mammalian *ram* mutant by genetic manipulation of ribosomal protein S2. The mutation affects general translation by increasing the random misincorporation of near-cognate amino acids. Compared to HEK293 cells transfected with a wild-type *RPS2* gene and a transgene expression level of 30–40% of total *RPS2* mRNA, HEK293 cells transfected with the A226Y mutant *RPS2* transgene showed a reduced expression corresponding to ~10% of total *RPS2* mRNA (Fig. 1a). While the reduced expression of the mutant transgene likely reflects the fitness-associated cost of ribosomal misreading, our data demonstrate that even at this level of expression, the mutant RPS2 protein conferred significant ribosomal misreading as testified by misreading and read-through reporters (Table 1). Starting with a global transcriptomic approach, we profiled and further characterized the mammalian response towards chronic mistranslation. Chronic mistranslation led to a proteostatic response that attenuated cytosolic protein synthesis and the cell cycle, while increasing expression of chaperones and the ubiquitin-proteasome system to prevent accumulation of misfolded proteins. Pointing to increased proteolytic activity as an adaptive response towards ribosomal mistranslation, we found that *RPS2* A226Y mutant cells show increased susceptibility to treatment with the proteasomal inhibitor MG132. Activation of proteasome-mediated proteolysis was accompanied by increased recycling of amino acids.

Rapid attenuation of global protein synthesis under protein stress has been largely attributed to the inhibition of translation initiation^43^. Here, we found little or no evidence of initiation inhibition. Instead, we found that in addition to decreased transcript levels of genes involved in cytosolic protein synthesis the reduced translational activity observed in the *RPS2* A226Y mutant is associated with elongation slowdown. This finding is supported by a recent report on the fine-tuning of ribosomal elongation in response to proteotoxic stress^44^.

An important contributor of the UPR^ER^, often left unnoticed, is the regulation of protein transport across the ER membrane. Here, we showed that mRNA transcripts representing all three major pathways for ER import were decreased in the mutant. Further transcriptomic changes suggest that ER-destined proteins are rerouted to an alternative pathway for proteolytic digestion using the MLP quality control machinery. We used an ER-luciferase reporter to directly demonstrate that protein import into the ER was reduced in the A226Y mutant. We compared our findings to a study using classical UPR^ER^ inducing agent tunicamycin^45^. Tunicamycin showed a completely opposite transcriptomic response, upregulating the expression of all ER translocation machineries (Supplementary Fig. 9). In contrast to tunicamycin, which creates ER stress by inhibiting glycosylation of export-bound glycoproteins within the ER, the A226Y mutants affect the ER at a pre-ER stage, i.e., by increasing the error rate of protein synthesis. On the basis of our observations, we hypothesize that limiting ER protein translocation shields the ER from import of mistranslated proteins, thus preventing the ER from an overload of proteins which would trigger apoptosis through prolonged activation of the UPR^ER^ stress sensors. In addition to shielding the ER by limiting protein translocation, we found increased gene transcripts for the small GTPase Sar1 which controls ER export and facilitates trafficking of misfolded ER proteins to the Golgi^46,47^.

Our data reveal an unexpected downregulation of the UPR^ER^ pathway in the A226Y mutants. Supported by heightened susceptibility to p53-mediated cell death in the A226Y mutants, our findings suggest that the downregulation of UPR^ER^ may aid in circumventing UPR^ER^ triggered apoptosis. The finding that chronic ribosomal mistranslation triggers silencing of the UPR^ER^ was unexpected, as previous reports using classical UPR inducers concluded that under conditions of unresolved ER stress, terminal UPR^ER^ is activated, resulting in cell death^48^. How chronic mistranslation silences UPR^ER^ to avoid triggering UPR-mediated apoptosis remains a subject for future investigation.

In sharp contrast to limiting protein import into the ER, we observed a significant activation of mitochondrial import, biogenesis and increased mitophagy in the A226Y mutants. These findings are mirrored by treatment with proteasome inhibitors and aminoglycosides, known misreading agents. Thus, proteotoxic stress induced either by increasing ribosomal mistranslation or by inhibiting proteasomal degradation, lead both to an increase in mitochondrial import, biogenesis and mitophagy. The importance of mitochondria in the cell’s response to increased protein misfolding is supported by the heightened susceptibility of the A226Y mutants to CCCP, a disruptor of the mitochondrial membrane potential required for protein import.

The progressive loss of proteostasis observed during aging and neurodegenerative diseases has been characterized by the accumulation of protein aggregates and mitochondrial dysfunction^49,50^. In addition, aggregation-prone markers of neurodegenerative diseases like the amyloid β-peptide have curiously been found to accumulate in the mitochondria^51,52^. Thus far, it remained unclear how mitochondrial dysfunction and protein aggregation could be related. It was recently shown in yeast that aggregation of cytosolically misfolded proteins occurs in a partly organelle-based manner, facilitating asymmetric retention upon cell division and import into the mitochondria for degradation^40,53^. The import of protein aggregates was dependent on the mitochondrial import machinery and on yeast-only chaperone Hsp104, possibly to assist the unfolding of protein aggregates to enable entry via the Tom40 channel complex.

Using cell fractionation experiments and treatment with proteinase K with or without membrane-permeabilizing detergents in combination with functional enzymatic activity assays and visualization by means of the split GFP system, we directly demonstrate that cytosolically misfolded proteins localize to mitochondria in HEK293 cells, thus providing strong evidence for a role of mitochondria in proteostasis in mammalians. We hypothesize that aggregation-prone proteins enter the mitochondria for compartmentalization and subsequent degradation. Chronically, accumulation of misfolded proteins in the mitochondria may lead to mitochondrial dysfunction, as observed in the A226Y mutants. In addition, the slowdown of protein import into the ER as observed in the mistranslating A226Y mutants may promote mistargeting of proteins normally targeted to ER to the mitochondria^54,55^. While the mechanistic details require further investigation, we hypothesize that the increase in mitophagy of the A226Y mutants functions to selectively degrade dysfunctional mitochondria together with their proteostatic “load”. This offers an attractive pathomechanistic concept for loss-of-autophagy associated neurodegeneration in mice^56,57^ and for familial Parkinson’s disease^58,59^ associated with mutations in parkin, a ubiquitin ligase promoting mitophagy.

Our approach using global gene expression profiling to study the cellular response towards chronic ribosomal mistranslation has enabled us to characterize how various individual pathways work together and to uncover previously undescribed responses. Our data provide a framework for further elucidation of networks that contribute to protein homeostasis.

## Methods

### Vector construction and generation of stable transfectants

Expression vectors p*RPS2*-WT and p*RPS2*-A226Y were constructed by Genoway, France. Using 5′-BamHI and 3′-AvrII restriction sites, the mouse *Rps2* coding sequence [WT/A226Y (GCC→TAC)] was ligated into a proprietary eukaryotic expression vector which in addition to the CAGGS promoter carries the human growth hormone polyadenylation signal. The amino acid sequences of mouse and human Rps2 are identical. The mouse gene was chosen, because the difference in nucleic acid sequences between human and mouse RPS2 allows for precise quantification of transfected mouse RPS2 mRNA and endogenous human RPS2 mRNA. For visualization of target gene expression RPS2 was linked to IRES-eGFP. In addition, a Hyg^R^ cassette from plasmid pGL4.14 (Promega) was inserted at the MluI restriction site. HEK293 cells were transfected with p*RPS2* (WT/A226Y) expression vectors using Turbofect (Thermo Fisher Scientific) and cultured in DMEM with 10% FBS under 100 µg/ml hygromycin B selection for 5–7 weeks. GFP expressing colonies were picked for further characterization. GFP fluorescence was analyzed by flow cytometry using the BD FACS Canto II (BD Biosciences) and the FlowJo software (Tree Star Inc). Several independent clones from each pRps2-WT and pRps2-A226Y transfected cells were used for all further experiments; the number of independent clones used in each experiment is specified in the corresponding figure legend.

Plasmids MTS-mCherry-GFP1–10, FlucWT-HA-GFP11, and FlucDM-HA-GFP11 were obtained from Addgene, USA^40^. Neo^R^ cassette in vectors FlucWT-HA-GFP11 and FlucDM-HA-GFP11 was replaced with Amp^R^ and Hyg^R^ cassettes from vector pGL4.14 (Promega) using 5′-Stul and 3′-Pcil restriction sites resulting in vectors FlucWT-HA-GFP11-hyg and FlucDM-HA-GFP11-hyg.

### Determination of *RPS2* mRNA expression

mRNA was isolated using TRIzol (Thermo Fischer). qRT-PCR was used to determine the relative ratio of *RPS2* transgene mRNA to endogenous *RPS2* mRNA using RPS2 primers flanking the site of mutation. Discrimination was achieved using TaqMan probes specific for human wild-type endogenous *RPS2* (5′-CAC CTC AGC CCG GG-3′, VIC), wild-type mouse transgene (5′-TGC TAC ACT TCA GCC-3′, NED) or mutant mouse transgene (5′-CTA CAC TTC ATA CAG AG-3′ for A226Y, FAM). Experiments were conducted in triplicates using the TaqMan Kit (Life Technologies) and the ABI 7500 Fast Real-Time PCR System (Life Technologies). Amplification of 40 cycles (95 °C for 20 s and 60 °C for 45 s); the ratio of transgene versus endogenous *RPS2* was calculated as previously described^60^.

### Detection of transfected RPS2 in polysome fractions

For cellular localization of the transgenic RPS2 protein, a myc-tag (EQKLISEEDLN) was introduced to the C-terminal of RPS2, and western blotting using anti-myc antibodies was performed.

For preparation of S100 and ribosome fractions the cell lysate prepared as in^61^ was diluted to 2 ml and adjusted to 0.5 M KCI. Cell lysates were loaded on a 1 M sucrose cushion in 3-ml polycarbonate tubes and ultracentrifuged for 2 h at 250,000 × *g* at 4 °C in a TL100.3 rotor (Beckman). The supernatant above the sucrose cushion was collected (S100 fraction). The pellet (ribosome fraction) was re-suspended in buffer (20 mM Hepes pH = 7.6, 100 mM KCI, 5 mM MgCI_2_, 1× protease inhibitor cocktail, EDTA free).

Polysome profiling was performed as previously described in ref. ^61^. Five OD_260_ units were loaded onto a 5–50% sucrose gradient, ultracentifuged (SW41 rotor, 36,000 rpm, 4 °C) for 2 h, and fractionated using a Teledyne ISCO Polysome Profiler System. Fractions were collected and analyzed by western blot.

### Western blot and antibodies

Cells were grown to 70% confluency in DMEM with 10% FBS, lysed with 1× Passive Lysis Buffer (Promega) and ultrasonicated. Lysates were centrifuged (15,000 × *g*, 10 min), supernatant harvested and normalized to protein concentration as measured using Micro BCA Protein Assay Kit (Thermo Scientific). The specific antibodies used in this study were: polyclonal anti-myc (Abcam, ab9106); polyclonal anti-RPS2 (Abcam, ab154972); polyclonal anti-RPS9 (Abcam, ab117861); polyclonal anti-tubulin (Abcam, ab6046); polyclonal anti-alpha actin (Abcam, ab5694); polyclonal anti-peIF2α (Cell Signaling, 9721S); anti-eIF2α (Cell Signaling, 9722); polyclonal anti-ubiquitin (Abcam, ab7780); polyclonal anti-GAPDH (Abcam, ab9485); mouse monoclonal anti-p53 (Abcam, ab26); HRP-conjugated goat anti-rabbit (Invitrogen, G-21234); and goat anti-mouse antibodies (Invitrogen, A10551).

### Dual luciferase mistranslation reporter assay

Mistranslation was assessed as described previously^23,24^. Briefly, misreading was determined using the pRM hRluc-hFluc H245R vector, where His245 (CAC codon) was replaced by Arg245 (near-cognate CGC codon or non-cognate AGA codon). Read-through was determined using pRM hRluc-hFluc D357X, where Asp357 (GAC codon) was replaced by a UGA nonsense-codon in the firefly luciferase (Fluc) transcript. Cells were transfected using TurboFect (Thermo Fischer) according to manufacturer’s protocol. After 24 h incubation, cells were lysed and luminescence measured using the FLx800 luminometer (Bio-Tek Instruments). Renilla luciferase (rluc) activity was used as an internal control. Misreading and read-through were calculated by the ratio mutant Fluc/Rluc activity to wild-type Fluc/Rluc activity. For drug treatments, cells were transfected, incubated overnight, then treated with the drug for further 24 h prior to lysis and measurement of luciferase activity.

For the measurement of Hurler/CF-associated read-through, vector pRM hRluc-TGA-hFluc, which contains hRluc and hFluc separated by 27nt linker encoding for polypeptide STCDQPFGF with glutamine (Q) replaced by a UGA nonsense codon^23^ was modified as follows. Restriction sites EcoRI and XhoI were introduced to the 5′-end and 3′-end flanking the Rluc-Fluc linker sequence, resulting in vector pRM-hRluc-TGA2-hFluc. Vectors required for Hurler/CF-associated read-through were constructed by exchanging the linker sequence via the introduced EcoRI and XhoI restriction sites. Each linker included five allele-specific codons up-and downstream of the stop codon and restriction sites 5′-*XhoI* and 3′-*EcoRI*. TAG vector linker, 5′-**TCGAG/**TCG ACG TGC GAT TAG CCG TTC GGA TTC **G/AATT**-3′; Hurler disease context W392X linker^62^, 5′-**TCGAG/**GAT GGA GAA CAA CTC TAG GCA GAG GTC TCA AAG **G/AATT**-3′; Hurler disease context W402X linker^63^, 5′-**TCGAG/**GAT GAG GAG CAG CTC TAG GCC GAA GTG TCG CAG **G/AATT**-3′; Cystic fibrosis G542X linker^64^, 5′-**TCGAG/**GAC AAT ATA GTT CTT TGA GAA GGT GGA ATC ACA **G/AATT**-3′; Cystic fibrosis W1282X linker^65^, 5′-**TCGAG/**ATA ACT TTG CAA CAG TGA AGG AAA GCC TTT GGA **G/AATT**-3′. Subcloning of the inserts into pRM-hRluc-TGA2-hFluc backbone using XhoI and EcoRI restriction sites produced vectors pRM-hRluc-Hu-W392X-hFluc, pRM-hRluc-Hu-W402X-hFluc, pRM-hRluc-CF-G542X-hFluc, pRM-hRluc-CF-W1282X-hFluc, and pRM-hRluc-TAG-hFluc.

### Generation time assay

Cell growth was monitored using Alamar blue (Life Technologies). HEK293 cells were seeded on 24-well plates (BD Falcon) at low density and incubated in DMEM with 10% FBS at 37 °C. 10% Alamar blue (v/v) was added at time points 0, 24, 48, and 72 h and fluorescence was measured (excitation: 530 nm, emission: 590 nm) after 3 h incubation. Signal intensities at time point 0 h were set as one and growth curves were plotted. Generation time was calculated as *t*_D_ = ln(2)/slope.

### Firefly luciferase protein aggregation assay

pRM-hFluc was constructed by replacing hRluc in vector pGL4.75 (Promega) with Fluc from vector pGL4.14 (Promega). Cells were transiently transfected with pRM-hFluc. Forty-eight hours post transfection cells were resuspended in 1× Passive Lysis Buffer (Promega) at a 1:5 ratio for cell lysis and fractionated by centrifugation (16,000 × *g* for 25 min) into soluble and pellet fractions. The resulting fractions were analyzed by western blot using HRP-conjugated specific anti-Fluc antibodies (Abcam). The signal intensity of the Fluc bands in the different fractions was quantified using ImageQuant (GE Healthcare) and the ratio between pellet and soluble fractions was calculated.

### ER-firefly luciferase protein trafficking assay

Cells were transiently transfected with the ER-luciferase reporter vector pRM-PLN-hFluc-KDEL, carrying unmodified firefly (Fluc) luciferase with an N-terminal prolactine signal sequence for ER transport (MNIKGSPWKGSLLLLLVSNLLLCQSVAP) and a C-terminal KDEL retention sequence. Forty-eight hours post transfection cells were resuspended in MTE-P buffer (270 mM D-mannitol, 10 mM Tris base, 0.1 mM EDTA, pH = 7.4, supplemented with 1 mM PMSF) and homogenized using a Dounce homogenizer. Homogenates were centrifuged at 1400 × *g* for 10 min at 4 °C to remove cellular debris. An aliquot of supernatant was kept as total cellular protein fraction and the remaining supernatant was centrifuged at 15,000 × *g* for 10 min at 4 °C. The supernatant was loaded on a sterile 14 × 89–mm polyallomer ultracentrifuge tube (Beckman) containing a sucrose gradient (3 ml 1.3 M sucrose, 3 ml 1.5 M sucrose, 2 ml 2.0 M sucrose; 10 mM Tris base, 0.1 mM EDTA, pH = 7.6) and centrifuged at 152,000 × *g* for 70 min at 4 °C, acceleration/deceleration = 1. Using a 20-G needle and 1 ml syringe, the interface containing the ER was collected. The ER fraction was transferred to a 5.0 ml, 13 × 51 mm polyallomer ultracentrifuge tube (Beckman) and supplemented with additional ice-cold MTE-P to a final volume of 4 ml. Tubes were covered with parafilm and mixed by inversion until the suspension was homogeneous and ultracentrifuged at 126,000 × *g* for 45 min at 4 °C (36700 rpm, SW60Ti rotor). Supernatant was discarded and the pellet was resuspended in ice-cold MTE-P. The resulting fractions were analyzed by western blot using HRP-conjugated specific anti-Fluc antibodies (Abcam). The signal intensity of the Fluc bands in the different fractions was quantified using ImageQuant (GE Healthcare).

### Endo H treatment

Endo H glycosidase (Promega) treatment was done according to manufacturer’s protocol. Sixty microliter of total cellular protein extract were denatured at 95 °C for 5 min using denaturating solution, cooled down to room-temperature for 5 min, Endo H buffer (Promega) and 2000 units of Endo H glycosidase were added. Samples were incubated for 3 h at 37 °C. The resulting samples were analyzed by western blot using HRP-conjugated specific anti-Fluc antibodies (Abcam). The signal intensities of the Fluc bands were quantified using ImageQuant.

### In vivo cytoplasmic translation

HEK293 cells were grown in 24-well plates in DMEM with 10% FBS to 80% confluency, washed and incubated for 30 min in methionine-free RPMI. The media was replaced with methionine-free RPMI supplemented with 10 mCi of ^35^S-Methionine (Hartmann) and incubated at 37 °C for the indicated time periods. Translation was stopped by the addition of a stop medium (DMEM supplemented with 100 µg/ml cycloheximide and 1 mg/ml *cold* methionine). Cells were detached by pipetting and washed once with 1× PBS before lysing with 1× Passive Lysis Buffer (Promega). Proteins were precipitated with 10% TCA (Sigma). Precipitates were collected on GF/C filter membranes (Perkin-Elmer) and [^35^S] incorporation was measured by scintillation counting (Perkin Elmer Trilux MicroBeta 96-well format counter).

### Polysome extraction, profiling, and runoff assays

Polysome extractions were performed as previously described^61^. One OD_260_ unit was loaded onto a 8–50% sucrose gradient, ultra-centrifuged (SW41 rotor, 39,000 rpm, 4 °C) for 3 h, and analyzed using a Teledyne ISCO Polysome Profiler System. For the polysome runoff assay, cells were grown in 15 cm petri dishes to 80–90% confluency. The cells were detached using Accutase and resuspended in DMEM with 10% FBS. The cell suspension was incubated in a shaking incubator at 37 °C, 100 rpm for 1 h. At the start of the experiment, cells were pretreated with 1 µg/ml Harringtonine (Enzo) for different time points (*t* = 0 min, 2.5 min, and 5 min) and 100 µg/ml cycloheximide was added at the end point. The cells were kept on ice until they were pelleted at 500 × *g* for 5 min. Polysomes were analyzed as described above. Relative polysome/monosome (P/M) ratios were calculated and normalized to *t* = 0.

### Cell cycle analysis using propidium iodide staining

DNA staining with propidium iodide (Thermo Scientific) was used to allow distinction between G1, S, and G2/M phase cells. At ~80% confluency, cells seeded in 12-well plates were washed with PBS and trypsinized. The detached cells were resuspended in DMEM and centrifuged for 5 min at 500 × *g* at room temperature. The pellets were resuspended in 300 μl ice cold PBS. One mililiter of ice cold 70% ethanol was added to detached cells and incubated for at least 2 h at −20 °C for fixation. Next, cells were permeabilized by adding 350 μl of 0.05% Triton X-100 diluted in PBS (PBSX). After washing twice with PBS, cells were stained using 300 μl of staining solution (50 μg/ml propidium iodide, 20 μg/ml RNase A in PBS) for 30 min in the dark. Fixed and stained cells were analyzed by flow cytometry using the BD FACS Canto II (BD Biosciences) and the FlowJo data analysis software (Tree Star Inc).

### Cell cycle ELISA

Levels of cell cycle marker protein CDK2 protein phosphorylated TYR15 (CDK2-pTYR15) were determined using the Abcam Cell Cycle In-Cell ELISA Kit (ab140363) and performed according to manufacturer instructions.

### Proteasome activity

Proteasome activity was determined as previously described^66^. Briefly, cells were grown to 80% confluency and lysed using Passive Lysis Buffer (Promega). After centrifugation (16,000 × *g*, 10 min, 4 °C), protein concentration of the supernatant was determined using the Micro BCA Protein Assay Kit (Thermo Fischer). Activity assays were performed with 50 μg of total protein (200 μl total) in 96-well plates by adding 100 μM Suc-Leu-Leu-Val-Tyr-AMC peptide substrate (chemotrypsin-like activity; Bachem, I-1395) or 100 μM Ac–NIe–Pro–NIe–Asp–AMC (caspase-like activity; Bachem, I-1850). Fluorescence (excitation 380 nm, emission 460 nm) was measured every 5 min for 1 h at 25 °C using a microplate fluorometer Synergy H1 (BioTek).

### Analysis of UPR effectors by qPCR and XBP1-splicing assay

RNA from HEK293 cells was extracted using the ReliaPrep™ RNA Cell Miniprep Kit (Promega) and reverse transcribed using the High Capacity RNA-to-cDNA Kit (Applied Biosystems) according to manufacturer instructions. The 5× EvaGreen® QPCR Mix (Bio&Sell) was used together with the 7500 Fast Real-Time PCR System (Applied Biosystems). Primers used, ATF4: forward 5′-ACC TTC GAA TTA AGC ACA TTC CTC-3′, reverse 5′-GCC ACC TCC AGG TAA TCA TCT AAG-3′; ATF6: forward 5′-TTG GAA GCA GCA AAT GAG AC-3′, reverse 5′-GGA GAA AGT GGC TGA GGT TC-3′; XBP1: forward 5′-GGT CTG CTG AGT CCG CAG CAG G-3′, reverse 5′-GGG CTT GGT ATA TAT GTG G-3′; GRP94: forward 5′-TGG GAA GAG GTT CCA GAA TG-3′, reverse 5′-GTT GCC AGA CCA TCC GTA CT-3′; p53:forward 5′-GCG TGT GGA GTA TTT GGA TG-3′, reverse 5′-TGG TAC AGT CAG AGC CAA CC-3. GAPDH: forward 5′-ACC CAC TCC TCC ACC TTT GA-3′, reverse 5′-CTG TTG CTG TAG CCA AAT TCG T-3′. GAPDH expression was used as internal reference. Measured quantification cycles were analyzed according to Pfaffl^67^.

The XBP1-splicing assay was performed as previously described^23^.

### Caspase 3/7-assays and TUNEL-assays

Caspase 3/7 activity was measured using the ApoLive-Glo™ Multiplex Assay according to manufacturer instructions. The in situ BrdU-Red DNA Fragmentation (TUNEL) assay kit (Abcam, ab66110) was used to detect DNA fragmentation by flow cytometry and was performed according to manufacturer instructions. Drug-treatments (STA, 10 µM, 6 h; RITA, 1 µM, 12 h) for specific experiments were performed prior to paraformaldehyde fixing.

### Mitochondria staining, mtDNA:nDNA ratio determination

To estimate mitochondrial mass, Mitotracker™ DeepRed FM (M22426, Invitrogen) and TMRE (ab113852, Abcam) staining were performed according to manufacturer instructions and analyzed by flow cytometry using the BD FACS Canto II and the FlowJo software.

mtDNA:nDNA ratio was determined as previously described^38^. In brief, total DNA was extracted from cells with QIAamp DNA Mini Kit (Qiagen). For mtDNA, a fragment of the *MT-TL1* gene was amplified with forward primer, 5‘-CAC CCA AGA ACA GGG TTT GT-3‘ and reverse primer, 5‘-TGG CCA TGG GTA TGT TGT TA-3‘. For nDNA, a fragment of the *B2M* gene was amplified with forward primer, 5‘-TGC TGT CTC CAT GTT TGA TGT ATC T-3‘ and reverse primer, 5‘-TCT CTG CTC CCC ACC TCT AAG T-3‘. Amplification was performed in an ABI 7500 real-time PCR system (Life Technologies) for 40 cycles (95 °C for 20 s, 62 °C for 30 s).

### Basal cellular respiration

Mitochondrial respiration was measured using the Seahorse Bioscience XF24 analyser and XF24 microplates (Seahorse Bioscience) coated with 50 mg/ml poly-D-Lysine. ~10^5^ cells were seeded per well in 100 µl DMEM medium supplemented with 10% FBS, incubated for 3 h at 37 °C and 5% CO2 for cell attachment. Next, DMEM was aspirated and replaced with serum-free DMEM for overnight incubation. Prior to the experiment, the plate was incubated for 30 min at 37 °C in a CO2-free incubator. Cells were subsequently washed with Mitochondrial Assay Solution (MAS; 70 mM sucrose, 220 mM mannitol, 10 mM KH_2_PO, 4.5 mM MgCl_2_, 2 mM HEPES, 1 mM EGTA, 0.2% (w/v) fatty acid-free BSA, pH 7.2 at 37 °C) according to manufacturer instructions (Seahorse Bioscience). Measurements were performed in MAS, supplemented with 10 mM pyruvate, 10 mM succinate, 2 mM malate, and 0.2 nM Plasma Membrane Permeabilizer (PMP) reagent (Seahorse Bioscience). The oxygen consumption rate (OCR) in a coupled state (state 2, basal respiration) was measured. Data was extracted from the Seahorse XF-24 software and the bioenergetic parameters were calculated according to the manufacturer instructions.

### ATP and ROS measurements

ATP levels in HEK293 cell lines were determined using the ViaLight^TM^ plus kit (Lonza), which is based on luciferase activity. Five microliter Alamar blue (Life Technologies) were added to each well and incubated for 3 h. Alamar blue was used as an internal standard, followed by measurement of ATP content as per manufacturer instructions.

To detect mitochondria-specific superoxide in HEK293 cell lines, MitoSOX™ Red staining (Life Technologies) was performed in live cells. Cells were grown to ~80% confluency and stained with MitoSOX™ Red (5 μM) in FACS buffer (1× PBS, 2% FBS) for 30 min at 37 °C. Cells were detached using Accutase and resuspended in FACS buffer. Fluorescence was analyzed by flow cytometry using the BD FACS Canto II and the FlowJo data analysis software.

### In-vivo and in-organello mitochondrial translation

HEK293 cells were grown in DMEM plus 10% FBS in 24-well plates to 80% confluency. Cells were pre-incubated with methionine-free RPMI supplemented with 10% dialyzed FBS for 30 min, followed by 100 µg/ml Emetin for 10 min before the start of the experiment. 100 µg/ml Emetin was supplemented in all working media to suppress cytoplasmic translation. Cells were labeled by the addition of 20 mCi [^35^S] methionine (Hartmann) and incubated to the desired time points. Translation was stopped by the addition of 1 mg/ml cold methionine. Cells were detached by pipetting and washed once with 1× PBS before lysing with 1× Passive Lysis Buffer (Promega) supplemented with 0.1% SDS and 100 µU/µl DNaseI, incubated at 37 °C for 15 min followed by 10% TCA (Sigma) precipitation. The TCA precipitated cell extracts were collected on GF/C filter membranes (Perkin-Elmer) and [^35^S] incorporation was measured by scintillation counting (Perkin Elmer Trilux MicroBeta 96-well format counter).

Mitochondrial *in-organello* translation was done as previously described with slight modifications^68^. The mitochondria-enriched pellet was resuspended in 1 ml of mitochondria reaction buffer (MR-buffer) containing 15 μl of ^35^S-Methionine, 20 mM Tris-HCl (pH 7.2), 90 mM KCl, 4 mM MgSO_4_, 1.5 mM KH2PO_4_, 20 mM glutamate, 0.5 mM malate, 14 mM sucrose, 44 mM sorbitol, 4 mM ADP, 0.1 mM amino acids (without methionine), 1 mg/ml BSA (fatty acids free), and 0.1 mg/ml cycloheximide. *In-organello* translation was conducted by incubating at 30 °C for 2 h on a shaker (800 rpm). Cold methionine was subsequently added to a final concentration of 0.1 mM, reaction mixtures were centrifuged for 5 min at 15,000 × *g*, and supernatants were discarded. The pellets were washed with cold washing buffer containing 10 mM Tris-HCl (pH 7.4), 320 mM sucrose, 1 mM EDTA, centrifuged for 5 min at 15,000 × *g*, and resuspended in 20 μl of 1× SDS loading buffer. The samples were analyzed on 15% SDS PAGE. The gel was fixed, dried and exposed on phosphor-imager screen and scanned with laser scanner FLA-5100 (FUJI).

### Detection of mitophagy

To visually assess mitophagy, cells seeded on coverslips were fixed with 4% paraformaldehyde in PBS, and immunostained with anti-LAMP1 mouse monoclonal antibody (ab25630, Abcam) and anti-Tom20 rabbit polyclonal antibody (sc-11415, Santa Cruz Biotech). Goat anti mouse IgG AlexaFlour555 (ab150118, Abcam) and goat anti-rabbit IgG Cy5 (ab97077, Abcam) were used as secondary antibodies. Morphology and colocalization of mitochondria with lysosomes were assessed using a CLSM Leica SP5 Confocal Laser Scanning Inverted Microscope (Leica Microsystems) equipped with a helium neon laser. The objective used was oil immersion ×63/1.4. Plan Apo.

Colocalization of LAMP1 and TOM20 was quantified using Mander’s colocalization coefficient on ImageJ (COLOC2 plugin).

### Isolation of mitochondria for western blotting

Cells HEK293 were transiently transfected with the luciferase reporter vectors FlucWT-HA-GFP11 or FlucDM-HA-GFP11. Forty-eight hours post transfection cells were resuspended in MTE-P buffer (270 mM D-mannitol, 10 mM Tris base, 0.1 mM EDTA, pH = 7.4, supplemented with 1 mM PMSF) and homogenized using a Dounce homogenizer. Homogenates were centrifuged at 1400×*g* for 10 min at 4 °C to remove cellular debris. An aliquot of supernatant was kept as total cellular protein fraction. The remaining supernatant was centrifuged at 15,000×*g* for 10 min at 4 °C. The resulting supernatant was kept as cytosolic fraction. The resulting pellet was carefully washed twice with 0.5 ml of ice-cold MTE-P, resuspended in 0.8 ml of ice-cold MTE-P buffer and loaded slowly on top of a sucrose gradient (1 ml of 1.7 M sucrose overlaid with 1.6 ml of 1.0 M sucrose in a sterile 5.0 ml, 13 × 51 mm Beckman polyallomer ultracentrifuge tube). The tube was filled up with an additional 0.8 ml of ice-cold MTE-P buffer and centrifuged for 22 min at 40,000 × *g*, 4 °C (20600 rpm in an SW50.1 rotor). The interface between the 1.7 M and 1.0 M sucrose layers was collected (0.4 ml) and used as source of purified mitochondria. The mitochondria fraction was mixed with 1.1 ml of ice-cold MTE-P buffer and centrifuged for 10 min at 15,000×*g*, 4 °C. Pellet was resuspended in 100 μl of MTE buffer without PMSF and analyzed by western blotting as described.

### Proteinase K treatment and mitochondrial membrane permeabilization

Vector pRM-MTS-hFluc was constructed by PCR amplification of the MTS (Mitochondria Target Signal) fragment from the MTS-mCherry-GFP1–10 vector and subsequent subcloning into the pRM-hFluc vector. HEK293 cells were transiently transfected with pRM-hFluc or pRM-MTS-hFluc, 24 h post transfection mitochondrial fractions were prepared as described above and OD at 600 nm was measured. Mitochondrial fractions were adjusted to OD_600_ = 1.0–1.5 and 10 µl volumes were used per reaction.

Mitochondria were permeabilized with digitonin (0.05%) or Triton-X100 (2%). After 10 min incubation at 4 °C, proteinase K was added (200 µg/ml final concentration) and incubated for 2 h at 4 °C. Input control samples were kept with cold MTE buffer w/o detergents or proteinase K. Finally, 10 µg of insulin (I5500 Sigma) were added for protein co-precipitation, followed by TCA precipitation for 30 min at 4 °C (final concentration −20% of TCA). Following TCA precipitation samples were centrifuged at 21,000×*g* for 20 min at 4 °C, and the resulting pellets were dissolved in 30 µl of 1 M Tris-HCl pH = 7.5. Samples were analyzed by western blotting. Anti-Fluc antibody was used to visualize luciferase protein; anti-matrix COX1 antibody was used to control for mitochondrial integrity and loading.

### Split-GFP assay

For split-GFP assay HEK293 cells were transiently co-transfected with vectors MTS-mCherry-GFP1–10 and FlucWT-HA-GFP11-hyg or MTS-mCherry-GFP1-10 and FlucDM-HA-GFP11-hyg. Twenty-four and 48 h post transfection GFP and mCherry fluorescence intensities were assessed by flow cytometry using FACS LSR Fortessa (BD Biosciences) or by wide-field fluorescent microscopy using InCellAnalyser 2500HS (GE Life Sciences). For quantification of the FACS data, FlowJo analysis software was used. Expression levels of Fluc WT and Fluc DM were normalized by western blot (Fluc WT versus Fluc DM) or by luciferase activity (untreated versus geneticin treatment). GFP/mCherry ratio relative to Fluc expression level was calculated and used as a measure of Fluc mistargeted to mitochondria.

### Quantification of wide-field fluorescent microscopy data

Paraformaldehyde fixed cells on 12 mm cover slips were imaged on GE InCell Analyser 2500 DS spinning disc microscope using DAPI, AlexaFluor 488 (for GFP) and RFP (for mCherry) channels. For each sample 100 images were acquired covering in total around 2 × 10^**4**^ cells. After global threshold segmentation, cellular outlines were acquired and integrated intensities of GFP and mCherry inside individual cells were calculated (using CellProfiler 3.0 software)^69^. Further analysis was done in Microsoft Excel software: cell measurements were imported as individual data rows; manual threshold was applied for the filtering out extremely autofluorescent apoptotic cells and the background signal from untreated cells was subtracted. The results were presented as the ratio between integrated intensities of GFP and mCherry relative to luciferase activity. Each sample was independently reproduced and analyzed 3 times.

### Statistics and reproducibility

Several independent clones of each pRps2-WT and pRps2-A226Y transfected cells were used for the experiments; the number of independent clones used in each experiment is specified in the corresponding figure legend. Statistical analysis was performed with Prism 5.0 software. For all assays, except quantification of wide-field fluorescent microscopy, unpaired Student’s *t*-test was used to determine significant difference between analyzed samples. Significance of wide-field fluorescent microscopy quantification was calculated using Mann-Whitney test.

### RNA sequencing

For transcriptome analysis we used 4 independent clones of RPS2-WT transfected cells and 3 independent clones of RPS2-A226Y-transfected cells. RNA sequencing (RNAseq) was performed at the UZH/ETH Functional Genomics Center Zurich (FGCZ [http://www.fgcz.ch/]) according to the Illumina RNA sequencing protocol. Differential gene expression analysis between sample groups of interest was performed using the R/bioconductor package edgeR^70^ To analyze enrichment of genes in specific cellular functions, differential expressed genes were mapped to process networks using MetaCore (GeneGo, Thomson Reuters) and to known biological ontologies based on the GO project (http://www.geneontology.org/) using gene annotation tool Enrichr^71^. GO terms with adjusted *p*-value < 0.05 were subjected to REVIGO web page^72^ for removal of redundant GO terms from the results. For the transcriptome analysis of A226Y-induced mistranslation, three *RPS2* A226Y and four *RPS2* wild-type biological replicates were compared. For the analysis of aminoglycoside induced mistranslation, cells were treated with 16 µM geneticin in F10 medium supplemented with 15 µg/ml saponin for 32 h; four biological replicates were used.

### Metabolome analysis

For metabolome analysis we used 4 independent clones of RPS2-WT transfected cells and 4 independent clones of RPS2-A226Y-transfected cells. Clones were synchronized in DMEM without FBS for 24 h. Next, cells were incubated for 24 h in DMEM containing 10% FBS. Cells were detached using Accutase and washed three times in PBS. The dry cell pellets were snap frozen using liquid nitrogen and stored at −80 °C. Metabolome analysis was performed by Metabolon according to published methods^73^. Samples were prepared using proprietary organic and aqueous extractions in order to remove proteins and to recover maximal amounts of small molecules. The extracted samples were split in 5 parts-two for analysis by two separate reverse phase (RP)/UPLC-MS/MS methods with positive ion mode electrospray ionization (ESI), one for analysis by RP/UPLC-MS/MS with negative ion mode ESI, one for analysis by HILIC/UPLC-MS/MS with negative ion mode ESI, and one sample was reserved for backup. Metabolome data sets were analyzed by Between Group Analysis (BGA)^74^ using the made4 package (bioconductor.org) in R. Normalized MS intensity data were used for BGA analyses based on principal component analysis (PCA). BGA was applied to a data set consisting of 578 metabolites. Results of the BGA were visualized by a scatter plot for the first axis of the BGA.

### 3D modeling

Visualization using PyMol v.1.8 (Schrödinger, Inc.) based on published ribosome structures. Sources: *S. cerevisiae* ribosome PDB 4V7R; human cytosolic ribosome PDB 4V6X.

### Reporting summary

Further information on research design is available in the Nature Research Reporting Summary linked to this article.

## Supplementary information

Supplementary Information

Reporting Summary

Description of Additional Supplementary Items

Supplementary Data 1

Supplementary Data 2

## Data Availability

Transcriptomic data have been deposited in NCBI’s Gene Expression Omnibus and are accessible through GEO Series accession number GSE115153. Metabolomic data have been deposited in EMBL-EBI MetaboLights database and are accessible through accession number MTBLS106.
