# Supplementary material for: Ribosomal mistranslation leads to silencing of the unfolded protein response and increased mitochondrial biogenesis
Source: Commun Biol. 2019 Oct 17;2:381. doi: 10.1038/s42003-019-0626-9 (PMC6797716; doi:10.1038/s42003-019-0626-9)
Supplement: Supplementary file 1 — Supplementary Information [file 42003_2019_626_MOESM1_ESM.pdf]

Supplementary Figures

Supplementary Figure 1

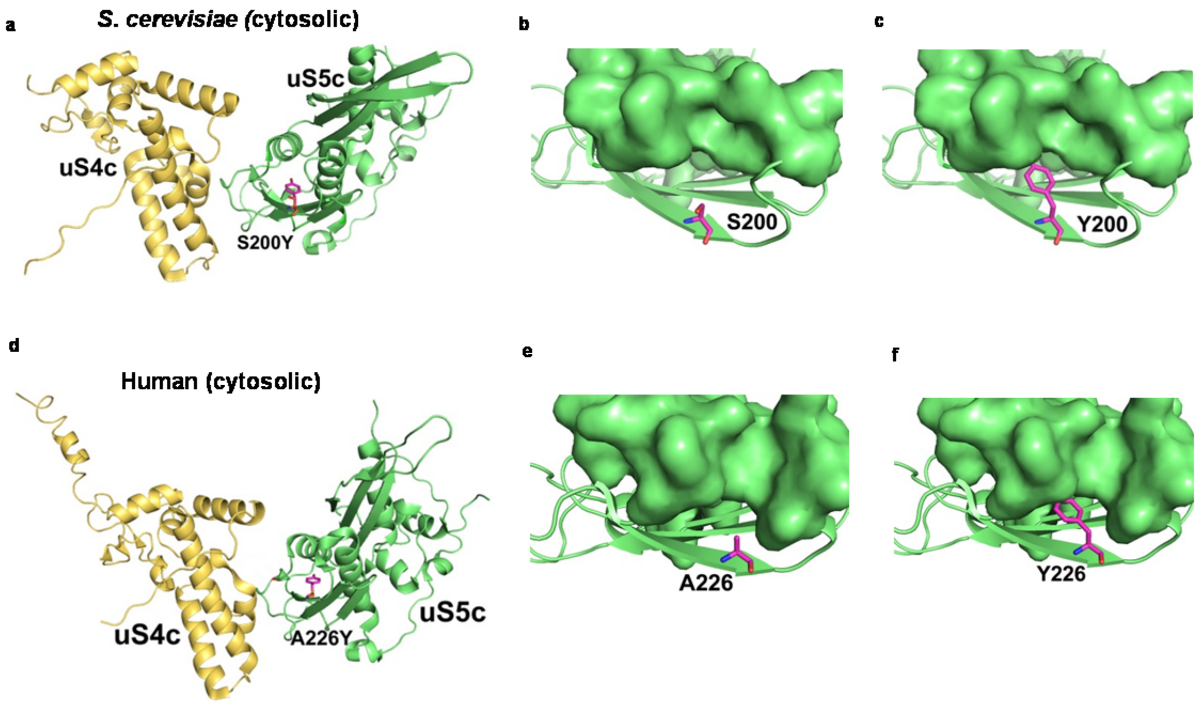

Modelling uS5 (RPS2) mutations at the uS4-uS5 (RPS9-RPS2) interface using PyMol v1.8 (Schrödinger Inc.) based on available crystal structures. uS4 (RPS9) and uS5 (RPS2) are shown in yellow and green respectively for all subsequent diagrams. (**a-c**) Modeling of S200 to Y200 substitution in uS5 of *S. cerevisiae.* (**a**) Protein-protein interface uS4-uS5 of the *S. cerevisiae* ribosome. Site of S200Y substitution is marked in magenta. (**b**) Position and orientation of native S200 residue. (**c**) Steric hindrance within the C-terminal domain of uS5 resulting from a S200 to Y200 substitution (Source – PDB 4V7R). (**d-f**) Modeling of A226Y substitution in uS5 of the human cytosolic ribosome. (**d**) Protein-protein interface uS4-uS5 of the human cytosolic ribosome. Site of S200Y substitution is marked in magenta. (**e, f**) Steric hindrance within the C-terminal domain of uS5 resulting from an A226 to Y226 substitution. (Source - PDB 4V6X).

Supplementary Figure 2

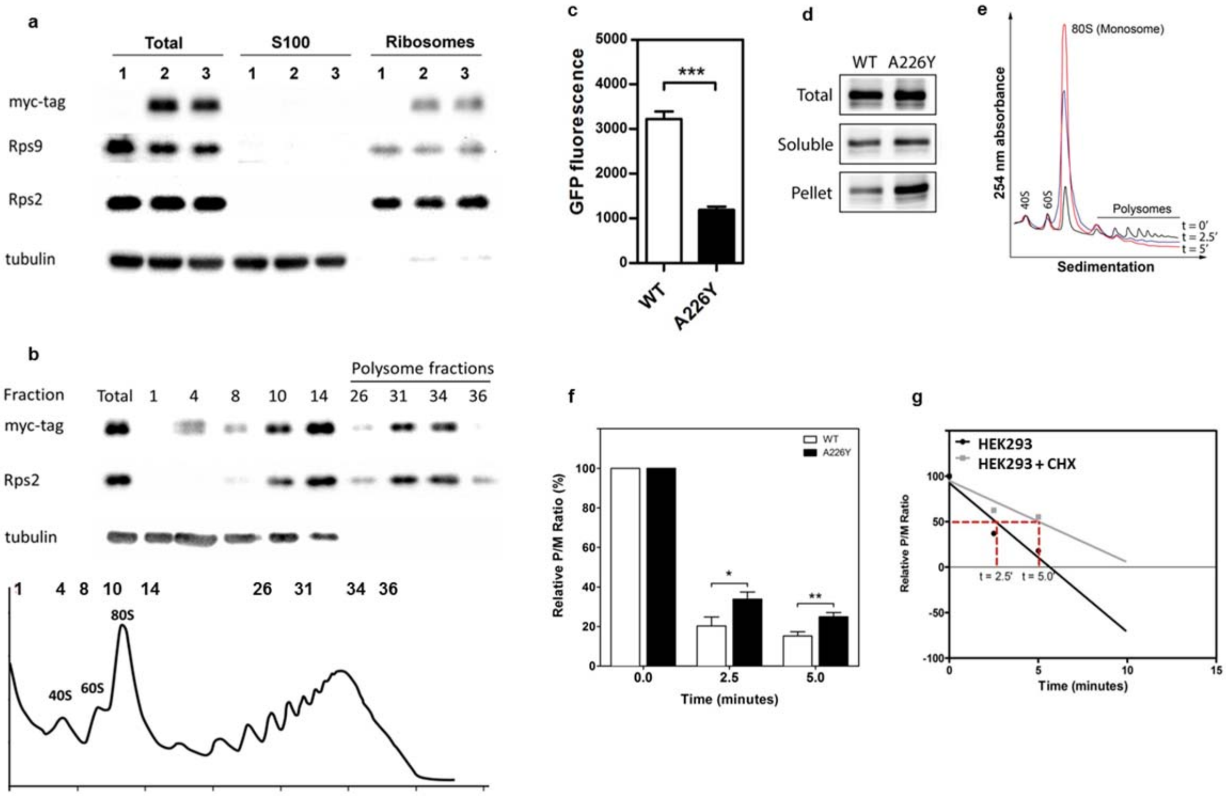

(**a**) Western blot showing enrichment of myc-tagged RPS2 transgenic protein in ribosomal fractions. HEK293 cells were transiently transfected using *RPS2 wt* and *RPS2* A226Y constructs. Lysates of whole cell, S100 extract (ribosome free) and ribosomal fractions were analyzed. Myc-tag antibodies were used for detection of transgenic RPS2. RPS2 antibodies were used to detect endogenous RPS2 protein. RPS9 antibodies were used to detect endogenous ribosomal protein RPS9, tubulin antibodies were used as a control for cytosolic protein. Lane 1: non-transfected HEK293 wt; lane 2: rps2 wt transfected; lane 3: rps2 A226Y transfected. (**b**) Western blot showing localization of myc-tagged RPS2 transgenic protein in ribosome and polysome fractions. HEK293 cells were transiently transfected using *RPS2* A226Y constructs. Lysates of whole cell and fractions from linear 5-50% sucrose density gradient were analyzed, fractions used for analysis are marked. Myc-tag antibodies were used for detection of transgenic RPS2, RPS2 antibodies were used to detect endogenous RPS2, tubulin antibodies were used as a control for cytosolic protein. (**c**) GFP fluorescence of HEK cells stably transfected with *RPS2* WT and *RPS2* A226Y as determined by FACS (N=6). (**d**) Representative Western blot of the firefly luciferase aggregation assay. Cell lysates were separated by centrifugation into soluble and insoluble fractions. The distribution of aggregation prone firefly luciferase shifts towards the insoluble pellet fraction in A226Y

mutants as detected using antibodies against firefly luciferase. (**e**) Gradual loss of polysomes following inhibition of translation initiation by harringtonine treatment, illustrated by the overlapping polysome profiles of HEK WT 0, 2.5 and 5 minutes post-harringtonine treatment. (**f**) The polysome/monosome (P/M) ratio normalized to t=0 for WT and A226Y for the polysome runoff assay (N=3). (**g**) Regression analysis of the P/M ratios of cycloheximide (CHX) treated cells plotted over time. CHX was used as a positive control for translation elongation slowdown. The polysome half-life is defined as the time it takes for each cell line to lose 50% of its polysomes following harringtonine treatment (illustrated as red dotted lines) (N=3). *$p<0.05$, **$p<0.01$, ***$p<0.005$. N = number of independent clones analysed for each genotype; for each clone 3 technical replicates were done. Mean ± SEM is given.

Supplementary Figure 3

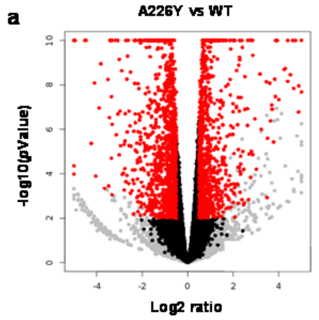

b

p-value	n	FDR	FC > 1.5	FC > 2
p < 0.05	5550	0.1259	2180	902
p < 0.01	3869	0.0361	1871	825
p < 0.001	2396	0.0057	1486	668

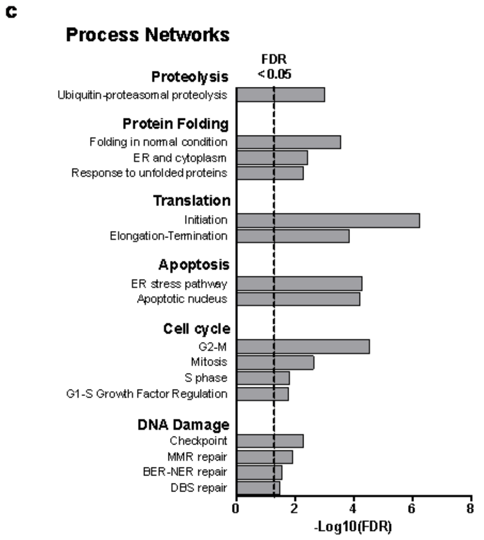

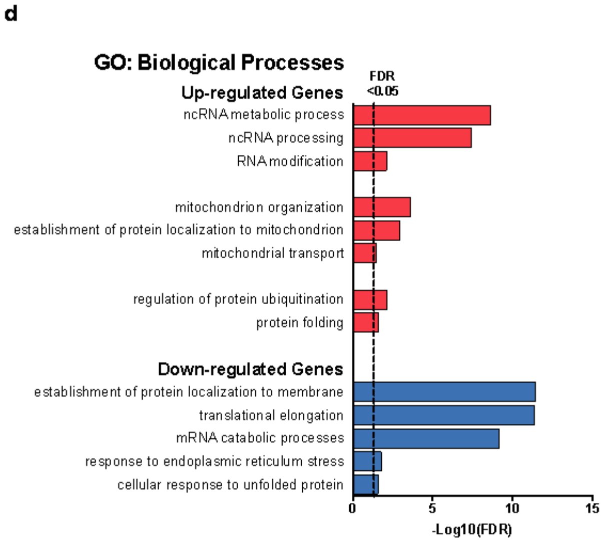

(**a**) Volcano plot summarizing the global transcriptome analysis performed by RNA-Seq (N≥3). The figure shows the fold change (log_2 ratio) and statistical significance [$-log_{10}$(pValue)] of all detected genes in A226Y *versus* WT. Genes differentially expressed by the A226Y mutants at a statistically significant level (p<0.01, log_2 ratio >0.5) are marked in red. Non-statistically significant genes are in black. (**b**) Table representing the number of differentially regulated genes (n) at different significance levels (p-value). Table also shows the false discovery rates (FDR) and the number of genes differentially expressed in A226Y at a 1.5- or 2-fold difference compared to WT (FC>1.5, FC>2) for each p-value cutoff. (**c**) Process network enrichment of A226Y mutants. 5550 genes (p<0.05) were submitted to Metacore from GeneGo for the analysis. Significantly enriched processes are shown for (i) proteolysis, (ii) protein folding, (iii) translation, (iv) apoptosis, (v) cell cycle and (vi) DNA damage pathways. (**d**) Biological Processes Enrichment of A226Y mutants. 5550 genes (p<0.05) were submitted to EnrichR for GO: biological processes enrichment analysis. Significantly enriched biological processes are shown for up- (red) and down-regulated

(blue) processes. For transcriptome analysis 4 independent clones of WT-transfected cells and 3 independent clones of A226Y-transfected cells were used.

Supplementary Figure 4

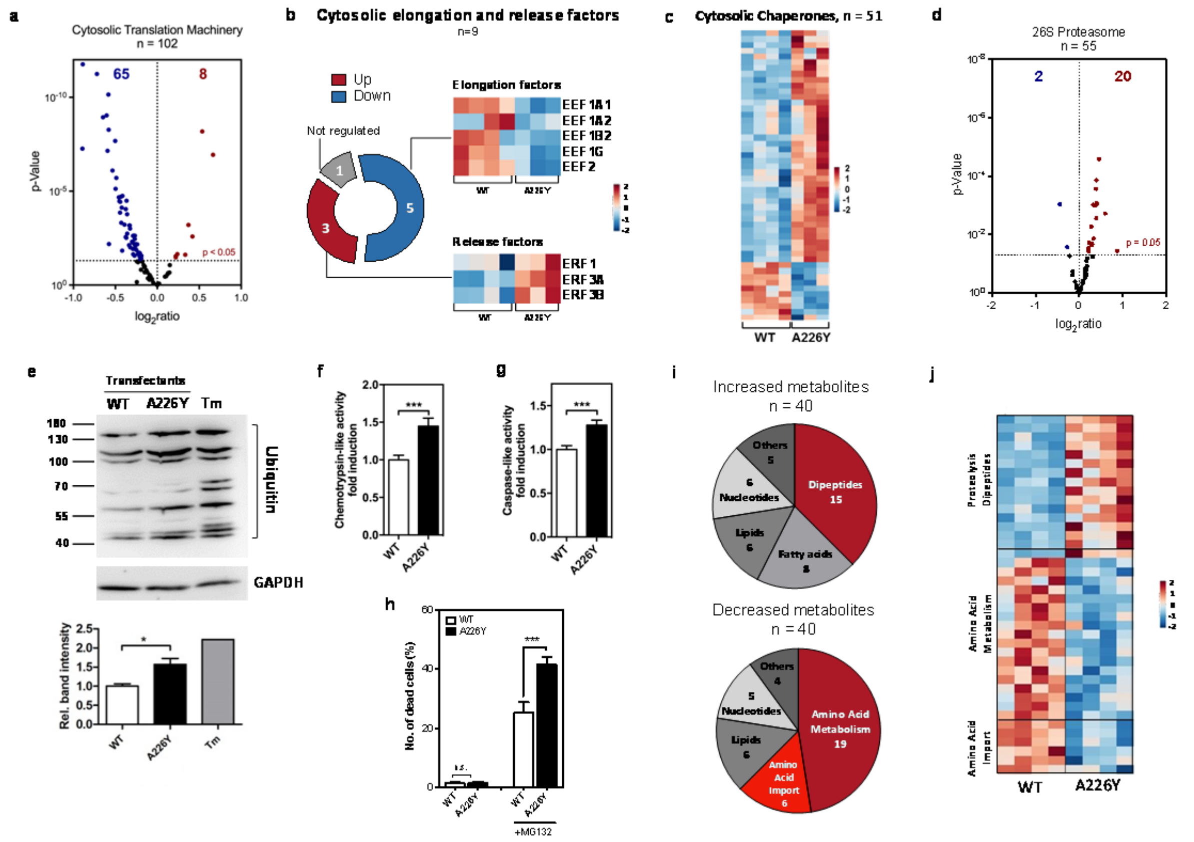

The response of the translational and proteolytic machinery in A226Y mutants. (**a**) Volcano plot of cytoplasmic ribosome structural genes and aminoacyl-tRNA synthetases (N≥3, $p<0.05$). (**b**) The pie chart shows the fraction of significantly regulated elongation and release factors (cytosolic) in the A226Y mutants ($p<0.05$). The heat maps illustrate how the elongation and release factors are regulated in consistent but opposite directions. (**c**) Heat map representing the significantly regulated cytosolic chaperones in the A226Y mutants ($p<0.05$). The full list was compiled from available literature and can be found in Supplementary Data 1[1]. (**d**) Volcano plot showing genes encoding the 26S proteasome (19S regulatory subunit, the 20S catalytic subunit and accessory factors). Blue dots represent significantly downregulated genes, red dots represent significantly upregulated genes (N≥3, $p<0.05$). The full list was compiled from available literature and can be found in Supplementary Data 1[2]. (**e**) Ubiquitination levels determined by Western blot. 20 µg of total protein were loaded and ubiquitin was detected by immunoblotting using specific antibodies. Tunicamycin (10 µg/ml, 4 h) used as positive control, GAPDH as loading control. A

representative blot is shown. Histogram represents the densitometric quantification of the ubiquitin bands (N=4). (**f, g**) Proteasomal activity as determined by measuring the chemotrypsin- and caspase-like activity of the proteolytic 20S core (N=4). (**h**) Significant increase in cell death for A226Y cells following inhibition of proteolytic activity by MG132 (20 µM, 4 h) as compared to WT cells. Cell viability was determined using Trypan blue (N=3). (**i, j**) A226Y mutant metabolome analysis (N=4, $p<0.05$). The top 40 metabolites which showed increased and decreased levels in A226Y mutants were extracted. (**i**) Pie charts showing the distribution of metabolites according to their pathway affiliation ($p<0.05$). (**j**) Heat map showing the metabolites involved in amino acid homeostasis, *i.e*. dipeptides, amino acid metabolism and amino acid import (*$p<0.05$; ***$p<0.005$). (**a-d**) For transcriptome analysis 4 independent clones of WT-transfected cells and 3 independent clones of A226Y-transfected cells were used; (**e-h**) N = number of independent clones analysed for each genotype; for each clone 3 technical replicates were done; (**i-j**) For metabolome analysis 4 independent clones of WT-transfected cells and 4 independent clones of A226Y-transfected cells were used. Mean ± SEM is given.

Supplementary Figure 5

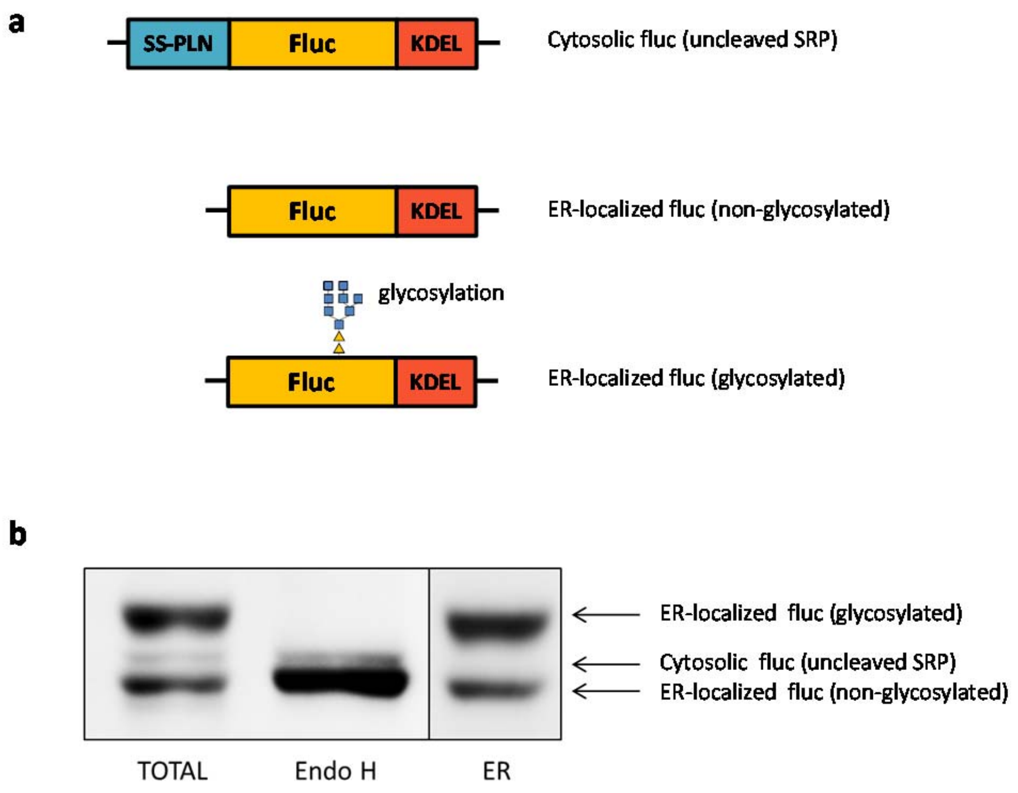

SRP-linked firefly luciferase localization assay. (**a**) Graphical representation of the three forms of Fluc. (**b**) Representative Western blot of HEK293 cells transfected with ER-Fluc. Total cell lysates of WT cells resolved by SDS-PAGE revealed three different luciferase forms using antibodies against firefly luciferase (Fluc). Upper band corresponds to ER-localised glycosylated Fluc; middle band corresponds to cytosolic Fluc still carrying the ER localization signal sequence (only cleaved upon entry into ER), lower band corresponds to ER-localised Fluc without signal sequence (cleaved but not glycosylated). The middle band represents Fluc not localized in the ER (mislocalized). Upon EndoH treatment, the upper band disappears as a result of the complete removal of the mannose modifications and is now incorporated into the cleaved fraction. In the ER isolated fraction, no middle band is detected as only the cleaved Flucs are present inside the ER.

Supplementary Figure 6

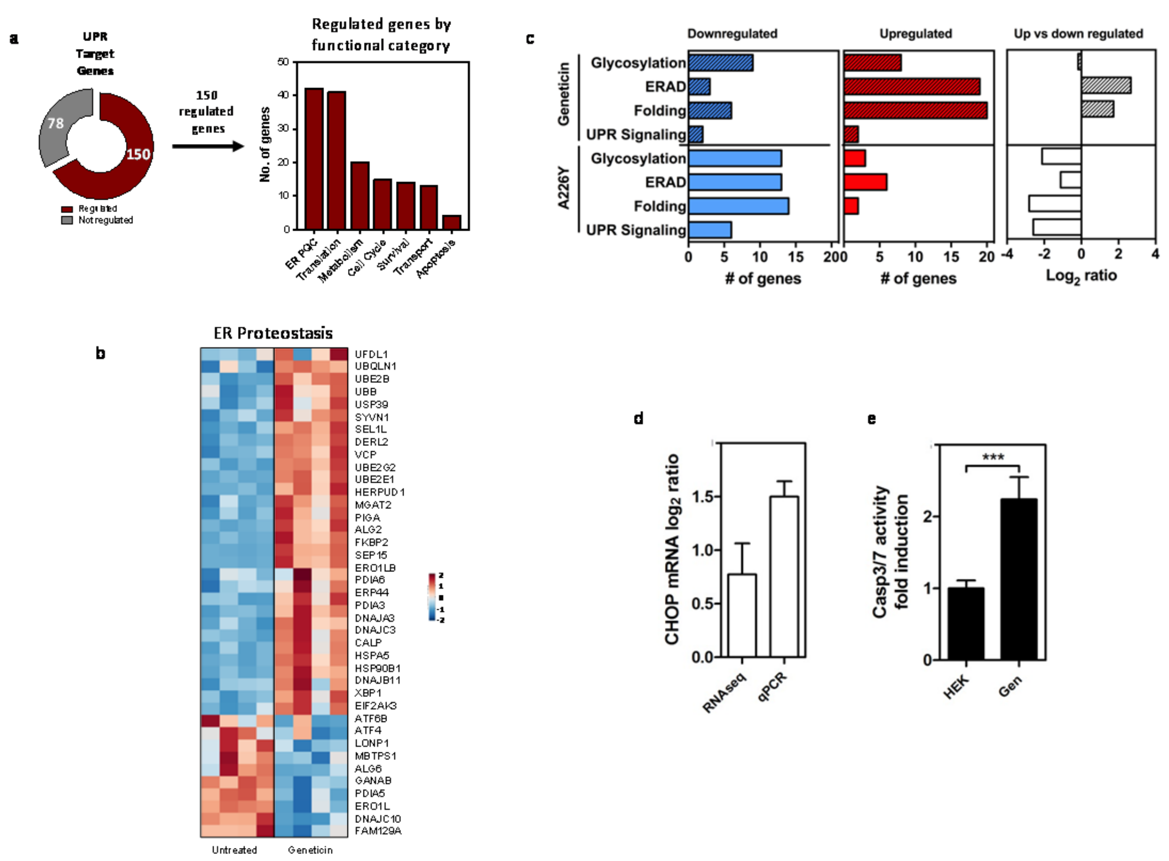

Geneticin-induced regulation of the ER unfolded protein response and apoptosis. (**a**) The pie chart represents the UPR target genes activated by transcription factors ATF6, XBP1 and ATF4 and the significantly regulated fraction (150/228) in geneticin treated cells ($p<0.05$). The histogram classifies the 150 regulated genes according to their functional categories. The full list of UPR target genes was compiled from available literature and can be found Supplementary Data 1[3-6]. (**b**) Heat map representing the significantly regulated UPR target genes ($p<0.05$) involved in ER protein quality control (ER-PQC) when treated with geneticin. (**c**) Side-by-side comparison of the UPR downstream response in A226Y mutants *versus* geneticin-treated HEK WT cells. The total number of regulated genes involved in N-linked glycosylation, ER-associated degradation (ERAD), ER folding machinery (Folding) and UPR signal transduction (UPR) ($p<0.05$) are represented. The far-right panel (Titled: Up *vs.* down regulated) shows the number of up- *versus* downregulated genes in each category as a ratio. This serves to indicate directionality in the regulation of these processes. The UPR response is clearly downregulated in A226Y as compared to geneticin-treated cells. (**d**) CHOP mRNA expression levels determined by RNAseq and qPCR (N=4). (**e**) Caspase 3/7

splicing activity assay. HEK293 wild-type cells treated with geneticin (16 μM, 72 h) *versus* untreated cells. Values were normalized to HEK-WT (N=3) (see Figure 4g for comparison). (**a-c**) For transcriptome analysis 4 independent samples of non-treated HEK293 cells and 4 independent samples of the cells treated with geneticin were used; (**d-e**) N = number of independent clones analysed for each genotype; for each clone 3 technical replicates were done. Mean ± SEM is given.

Supplementary Figure 7

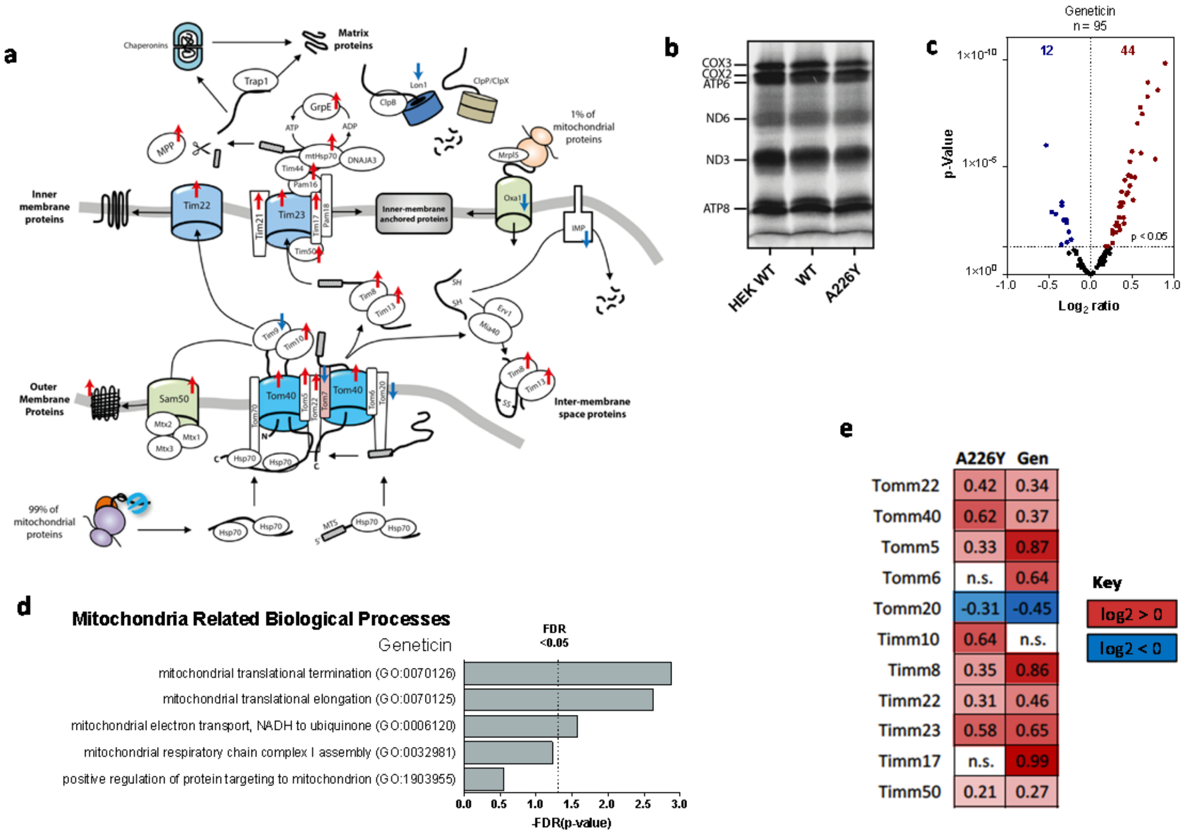

The mitochondrial response in A226Y mutants and geneticin-treated HEK293 cells. (**a**) The import pathway of mitochondria-bound proteins and its transcriptional response in the A226Y mutant. Red up-facing arrows (↑) indicate upregulation and blue down-facing arrows (↓) indicate downregulation. The full list of the mitochondrial import machinery was compiled from available literature and can be found in Supplementary Data 1[7]. (**b**) Representative autoradiograph of *in organello* translation using isolated mitochondria. (**c**) Volcano plots showing mRNAs for mitochondrial ribosomal proteins and aminoacyl tRNA synthetases in geneticin-treated HEK cells. Blue dots represent significantly downregulated genes while red dots represent significantly upregulated genes (N=4, p<0.05). (**d**) Significantly enriched mitochondria-related GO: biological processes in geneticin-treated cells (N=4, p<0.05). (**e**) Transcript heatmap of main mitochondrial import proteins for A226Y mutants (N=3, p<0.05) and geneticin-treated HEK cells (N=4, p<0.05). (**c-e**) N = number of independent samples treated with geneticin or independent transfected clones used for transcriptome analysis.

Supplementary Figure 8

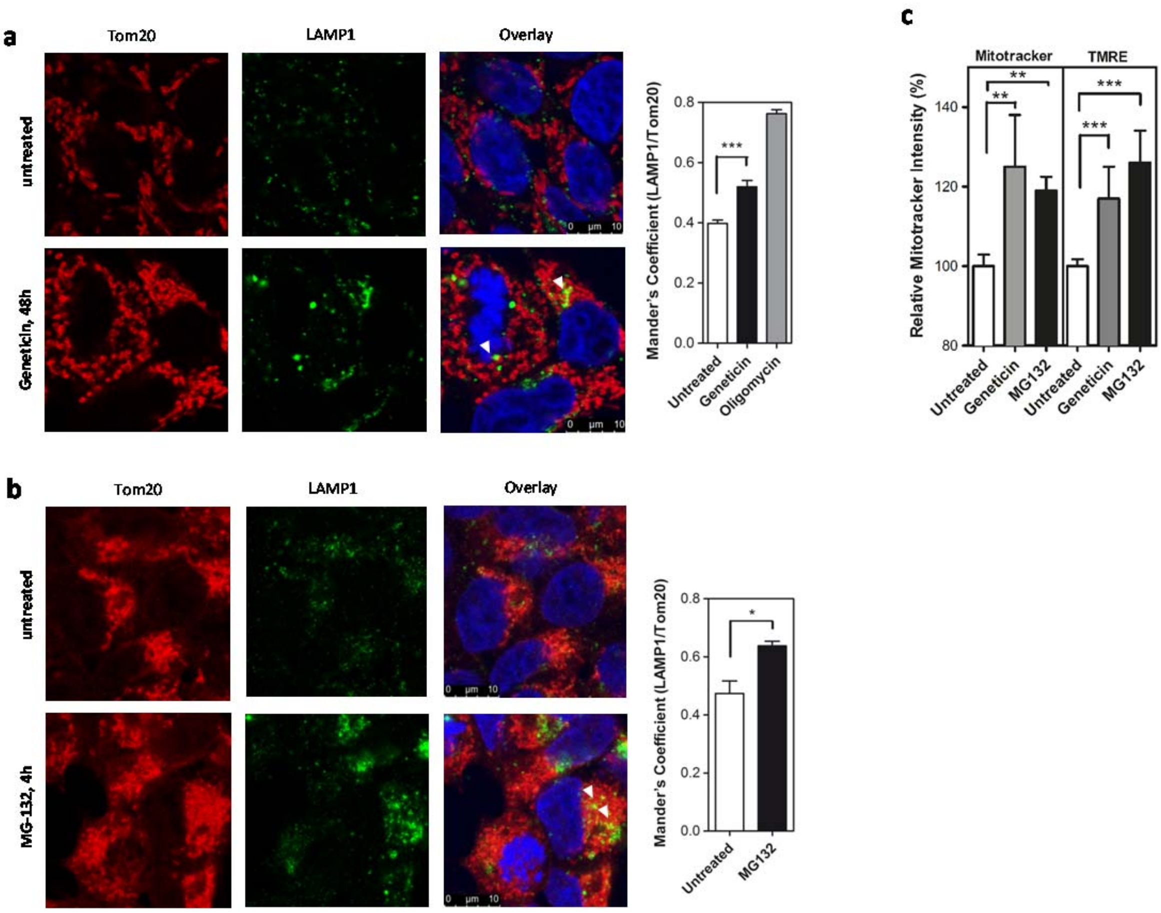

Increased mitochondrial mitophagy and mitomass in response to proteostatic stress. (**a**) Mitophagy in geneticin-treated HEK293 cells. Immunofluorescent images of mitochondria (Tom20, red) and lysosome (LAMP1, green). DAPI used to visualize cell nuclei (blue). Mitophagy is visualized by fusion of mitochondria with lysosomes (yellow) and marked with white triangles. Histogram represents the quantification of LAMP1 and Tom20 colocalization using Mander's Coefficient (N=5). Treatment of the cells with oligomycin (10 µM, 24 h) was used as a positive control for mitophagy[8]. (**b**) Mitophagy in MG132 (20 µM, 4 h) treated HEK293 cells. Immunofluorescent images of mitochondria (Tom20, red) and lysosome (LAMP1, green). DAPI used to visualize cell nuclei (blue). Mitophagy is visualized by fusion of mitochondria with lysosomes (yellow) and marked with white triangles. Histogram represents the quantification of LAMP1 and TOM20 colocalization using Mander's Coefficient (N=3). (**c**) Mitochondrial mass as measured by Mitotracker Deep Red FM and TMRE (FACS; mean fluorescence ± SEM; N=6). **p*<0.05, ***p*<0.01, ****p*<0.005. N = number of analysed independent samples treated with geneticin or MG-132; for each sample 2 technical replicates were done. Mean ± SEM is given.

Supplementary Figure 9

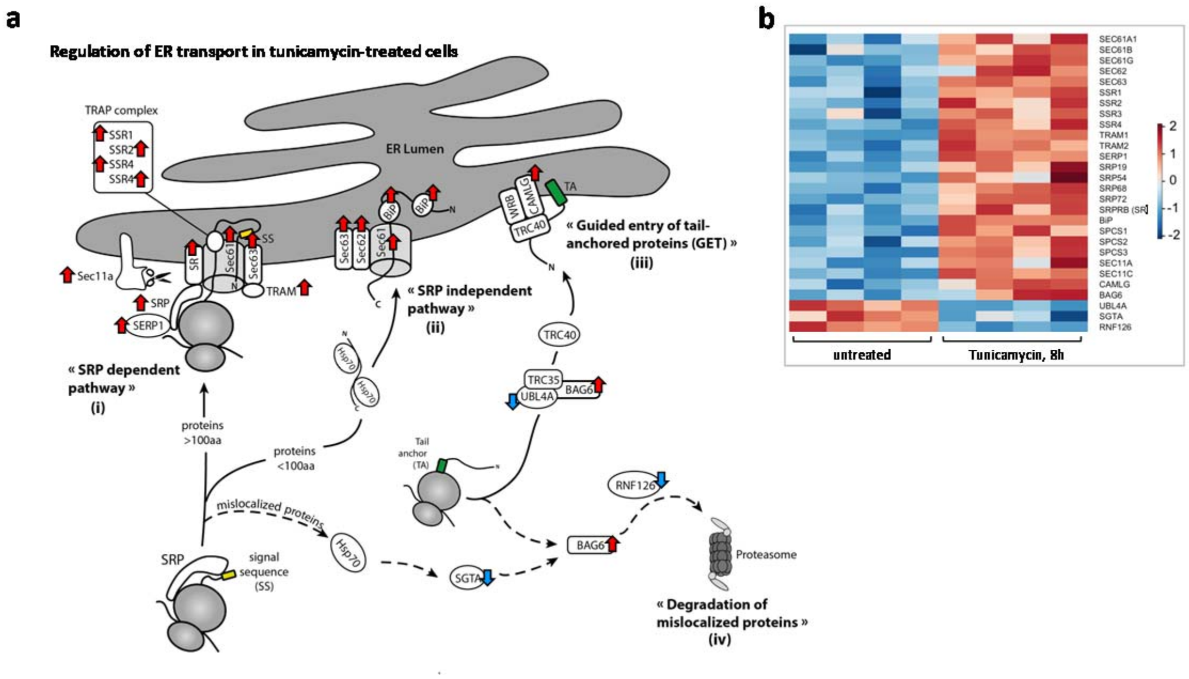

Regulation of ER transport in tunicamycin-treated cells. (**a**) ER-bound proteins are translocated across the ER membrane using either SRP-dependent or SRP-independent pathways. In contrast to A226Y mutant, genes involved in transport across the ER membrane are mostly upregulated in tunicamycin-treated cells (compare with Figure 2e). Red up-facing arrows (↑) indicate genes upregulated in tunicamycin-treated cells and blue down-facing arrows (↓) indicate genes downregulated in tunicamycin-treated cells. (**b**) Heatmap of the significantly regulated genes ($p<0.05$) involved in ER translocation from (a). Transcriptome data from this figure obtained from Oslowski and Urano, 2011. See main text discussion.

References

1. Saibil, H. Chaperone machines for protein folding, unfolding and disaggregation. *Nat Rev Mol Cell Biol* **14**, 630-642 (2013).
2. Ermolaeva, M. A., Dakhovnik, A. & Schumacher, B. Quality control mechanisms in cellular and systemic DNA damage responses. *Ageing Res Rev* **23**, 3-11 (2015).
3. Hebert, D. N. & Molinari, M. In and out of the ER: protein folding, quality control, degradation, and related human diseases. *Physiol Rev* **87**, 1377-1408 (2007).
4. Araki, K. & Nagata, K. Protein folding and quality control in the ER. *Cold Spring Harb Perspect Biol* **3**, a007526 (2011).
5. Lecca, M. R., Wagner, U., Patrignani, A., Berger, E. G. & Hennet, T. Genome-wide analysis of the unfolded protein response in fibroblasts from congenital disorders of glycosylation type-I patients. *Faseb J* **19**, 240-242 (2005).
6. Bernasconi, R. & Molinari, M. ERAD and ERAD tuning: disposal of cargo and of ERAD regulators from the mammalian ER. *Curr Opin Cell Biol* **23**, 176-183 (2011).
7. Becker, T., Bottinger, L. & Pfanner, N. Mitochondrial protein import: from transport pathways to an integrated network. *Trends Biochem Sci* **37**, 85-91 (2012).
8. Lazarou, M. *et al.* The ubiquitin kinase PINK1 recruits autophagy receptors to induce mitophagy. *Nature* **524**, 309-314 (2015).

Supplementary Gel Images

Supplementary Gel Image 1

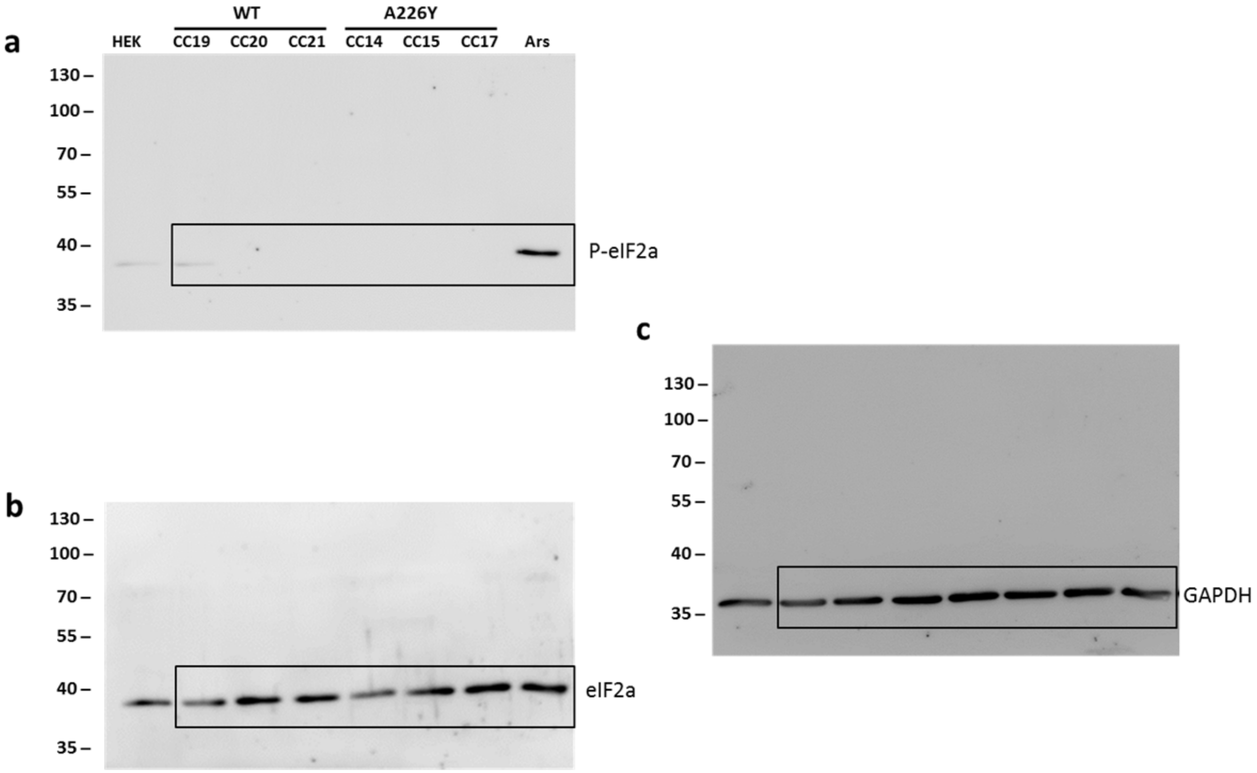

Figure 1g: full gel images with corresponding molecular size markers. Equal aliquots of samples were loaded onto three SDS gels, run in parallel and transferred onto three NC membranes. The membranes were stained with antibodies against phospho-eIF2a (**a**), eIF2a (**b**) or GAPDH (**c**) as a loading control and the bands were visualized with chemiluminescent detection system (see Methods).

Supplementary Gel Image 2

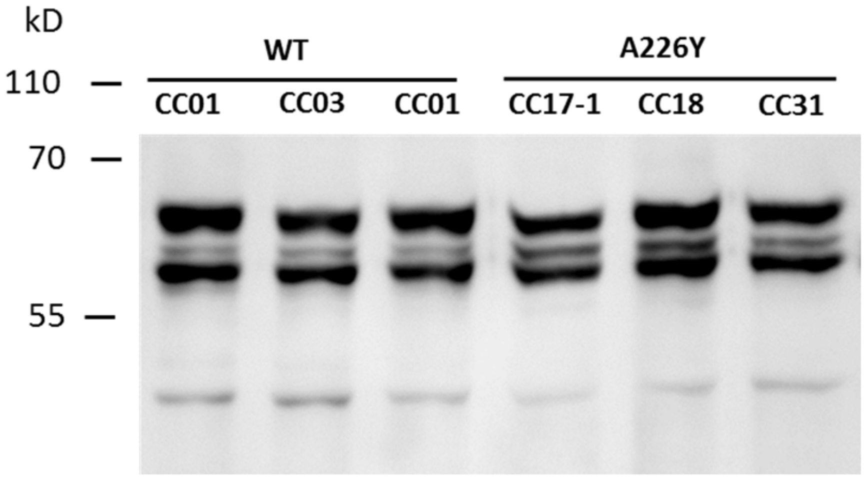

Figure 2f: full gel image with corresponding molecular size markers. Samples were loaded onto an SDS gel, run and transferred onto a NC membrane. The samples were stained with anti-Fluc antibody and the bands were visualized with chemiluminescent detection system (see Methods).

Supplementary Gel Image 3

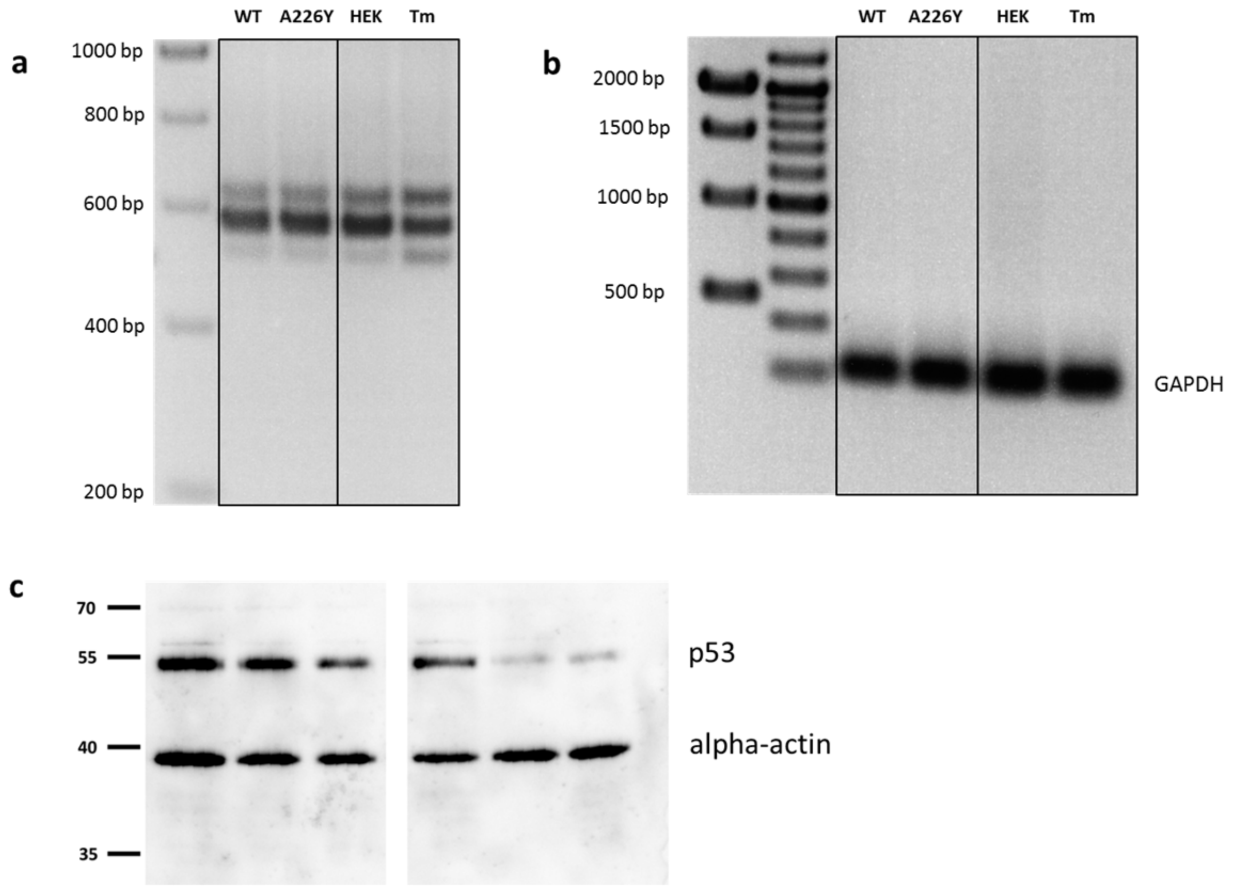

(**a**) and (**b**) – Figure 3c: full gel images with corresponding molecular size markers. Agarose gel analysis of the PCR products from XBP1-splicing assay (see Methods). Amplification of glyceraldehyde 3-phosphate dehydrogenase (GAPDH) cDNA served as a loading control.

(**c**) – Figure 3g: full gel images with corresponding molecular size markers. Equal aliquots of samples were loaded onto an SDS gel, run and transferred onto a NC membrane. The membrane was simultaneously stained with antibodies against p53 and alpha-actin as a loading control and the bands were visualized with chemiluminescent detection system (see Methods).

Supplementary Gel Image 4

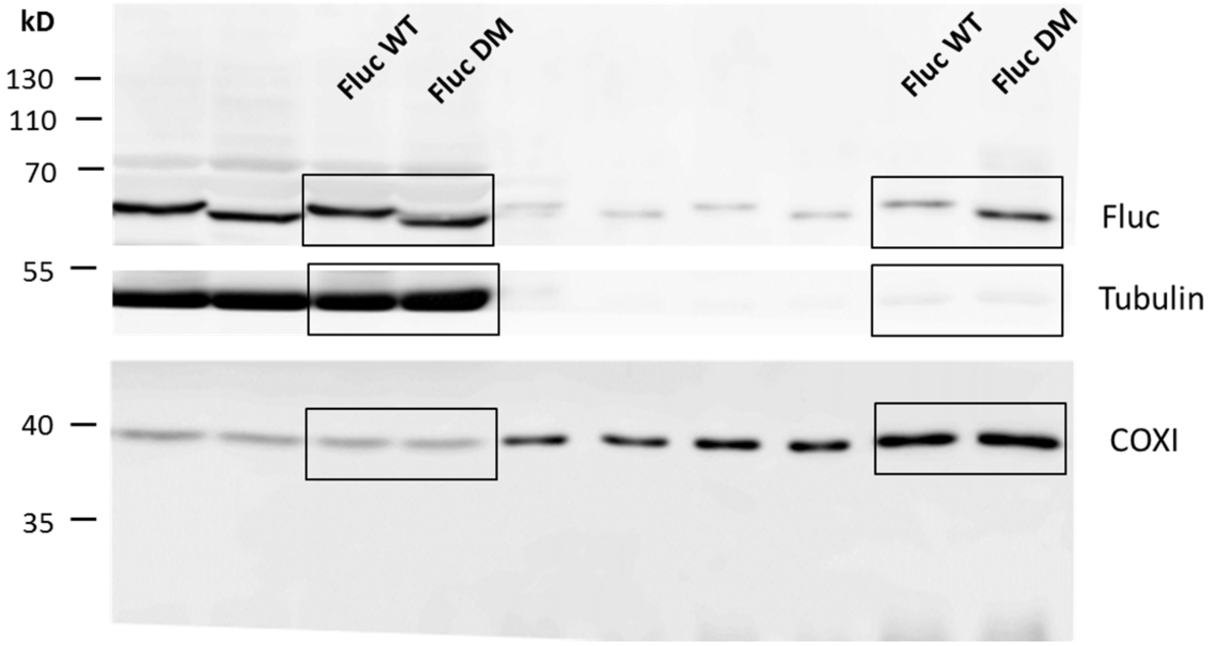

Figure 5a: full gel image with corresponding molecular size markers. Samples were loaded onto SDS gel, run and transferred onto NC membrane. The membrane was cut in 3 pieces according to molecular marker (areas >60 kD; 60-45 kD; <45 kD) and the pieces were separately stained with antibodies against Fluc, tubulin or COX1. The bands were visualized with chemiluminescent detection system (see Methods).

Supplementary Gel Image 5

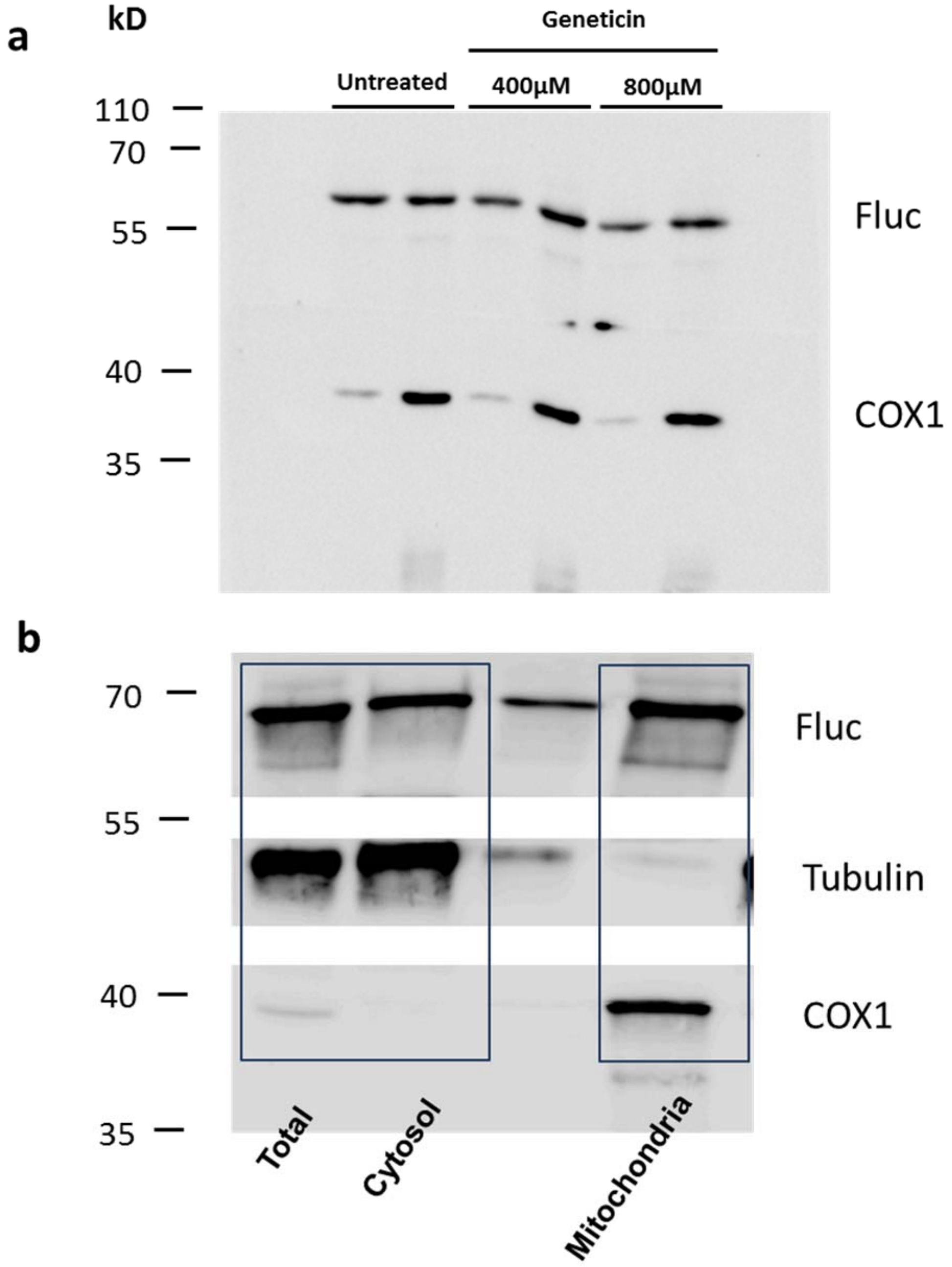

(**a**) – Figure 5c: full gel image with corresponding molecular size markers. The samples were loaded onto an SDS gel, run and transferred onto a NC membrane. The membrane was simultaneously stained with antibodies against Fluc and COX1 and the bands were visualized with chemiluminescent detection system (see Methods).

(**b**) – Figure 5e: full gel image with corresponding molecular size markers. Samples were loaded onto an SDS gel, run and transferred onto a NC membrane. The membrane was cut in 3 pieces according to molecular marker (areas >60 kD; 60-45 kD; <45 kD) and the pieces were separately stained with antibodies against Fluc, tubulin or COX1. The bands were visualized with chemiluminescent detection system (see Methods).

Supplementary Gel Image 6

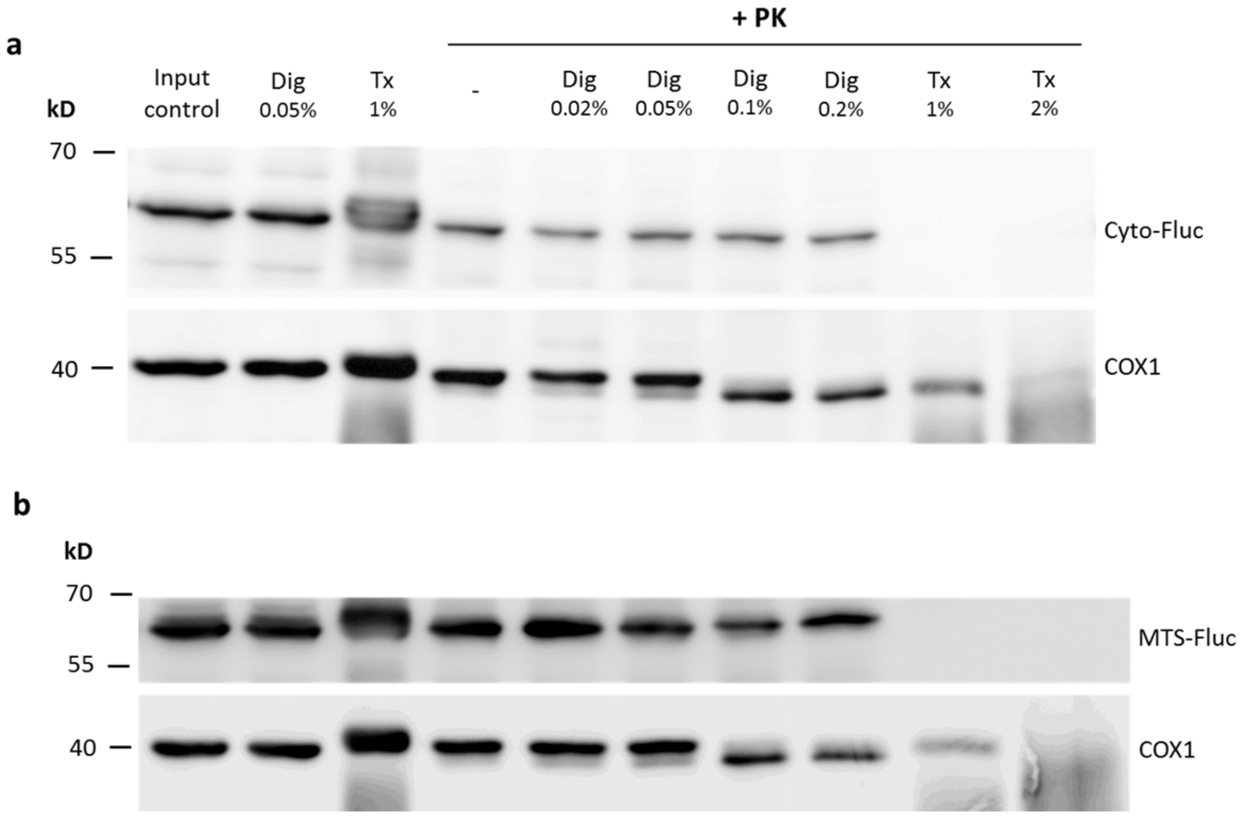

(**a**) – Figure 5g and (**b**) – figure 5h: full gel images with corresponding molecular size markers. Samples were loaded onto SDS gels, run and transferred onto NC membranes. The membranes were cut in 2 pieces according to molecular marker (areas >50 kD and <50 kD) and the pieces were separately stained with antibodies against Fluc or COX1. The bands were visualized with chemiluminescent detection system (see Methods).

Supplementary Tables

Supplementary Table 1

Top 40 Increased Metabolites in A226Y-induced Mistranslation			
**Metabolite**	**Pathway**	**Fold Change**	**Welch's ttest**
Phenylalanylalanine	Dipeptide	1.8	0.0030
Succinylcarnitine	TCA Cycle	6.2	0.0040
3-hydroxybutyrylcarnitine (1)	Fatty Acid Metabolism(Acyl Carnitine)	3.6	0.0067
Alanylleucine	Dipeptide	2.1	0.0118
Benzoylcarnitine*	Chemical	2.7	0.0076
Glutaminylleucine	Dipeptide	2.5	0.0065
15-methylpalmitate	Fatty Acid, Branched	1.3	0.0082
Isoleucylglycine	Dipeptide	1.3	0.0094
17-methylstearate	Fatty Acid, Branched	1.4	0.0191
Valylglycine	Dipeptide	2.0	0.0257
Histidylalanine	Dipeptide	1.8	0.0236
Nervonate (24:1n9)*	Long Chain Fatty Acid	3.3	0.0164
Tricosenoate (23:1)	Long Chain Fatty Acid	1.4	0.0124
Valylglutamine	Dipeptide	2.2	0.0248
Tyrosylglycine	Dipeptide	1.3	0.0108
Valylleucine	Dipeptide	3.4	0.0296
Phenylalanylglycine	Dipeptide	2.0	0.0370
Isovalerylcarnitine	Leucine, Isoleucine and Valine Metabolism	3.4	0.0373
Glycylisoleucine	Dipeptide	1.5	0.0173
Glycylvaline	Dipeptide	1.3	0.0250
Leucylglycine	Dipeptide	3.1	0.0497
Adenosine 2'-monophosphate (2'-AMP)	Purine Metabolism, Adenine containing	5.4	0.0483
Hexanoylcarnitine	Fatty Acid Metabolism(Acyl Carnitine)	4.3	0.0495
Glycerophosphoethanolamine	Phospholipid Metabolism	3.2	0.0257
N2-methylguanosine	Purine Metabolism, Guanine containing	1.3	0.0308
Sedoheptulose-7-phosphate	Pentose Phosphate Pathway	1.4	0.0247
1-(1-enyl-oleoyl)-2-oleoyl-GPE (P-18:1/18:1)*	Lysoplasmalogen	1.3	0.0353
Palmitoyl sphingomyelin (d18:1/16:0)	Sphingolipid Metabolism	1.2	0.0267
Threonylphenylalanine	Dipeptide	2.6	0.0495
3-hydroxybutyrate (BHBA)	Ketone Bodies	1.9	0.0252
Nonadecanoate (19:0)	Long Chain Fatty Acid	1.3	0.0511
2'-deoxyuridine	Pyrimidine Metabolism, Uracil containing	4.6	0.0637
5,6-dihydrothymine	Pyrimidine Metabolism, Thymine containing	1.9	0.0642
Cytidine 5'-diphosphocholine	Phospholipid Metabolism	1.8	0.0533
Carnitine	Carnitine Metabolism	1.5	0.0639
4-methylcatechol sulfate	Benzoate Metabolism	1.2	0.0329
3-aminoisobutyrate	Pyrimidine Metabolism, Thymine containing	1.8	0.0380

Leucylglutamine*	Dipeptide	2.4	0.0706
3-hydroxybutyrylcarnitine (2)	Fatty Acid Metabolism(Acyl Carnitine)	6.9	0.0743
Pseudouridine	Pyrimidine Metabolism, Uracil containing	1.3	0.0463

Supplementary Table 2

Top 40 Decreased Metabolites in A226Y-induced Mistranslation			
**Metabolite**	**Pathway**	**Fold Change**	**Welch's ttest**
Methionine	Methionine, Cysteine, SAM and Taurine Metabolism	0.8	0.0031
Nicotinamide adenine dinucleotide reduced (NADH)	Nicotinate and Nicotinamide Metabolism	0.2	0.0105
Gamma-glutamylglutamate	Gamma-glutamyl Amino Acid	0.5	0.0031
N-acetyltyrosine	Phenylalanine and Tyrosine Metabolism	0.4	0.0026
Citrulline	Urea cycle; Arginine and Proline Metabolism	0.7	0.0031
Nicotinamide adenine dinucleotide (NAD+)	Nicotinate and Nicotinamide Metabolism	0.5	0.0049
Isovaleryl CoA	Leucine, Isoleucine and Valine Metabolism	0.1	0.0209
Gamma-glutamylthreonine*	Gamma-glutamyl Amino Acid	0.6	0.0054
Glutamate	Glutamate Metabolism	0.8	0.0057
Gamma-glutamylcysteine	Gamma-glutamyl Amino Acid	0.6	0.0102
Glycerol	Glycerolipid Metabolism	0.6	0.0257
Gamma-glutamylisoleucine*	Gamma-glutamyl Amino Acid	0.6	0.0203
N-acetylleucine	Leucine, Isoleucine and Valine Metabolism	0.7	0.0322
N-acetyl-glucosamine 1-phosphate	Aminosugar Metabolism	0.6	0.0171
Cytidine 5'-monophosphate (5'-CMP)	Pyrimidine Metabolism, Cytidine containing	0.8	0.0211
Methionine sulfoxide	Methionine, Cysteine, SAM and Taurine Metabolism	0.8	0.0177
Gamma-glutamyltyrosine	Gamma-glutamyl Amino Acid	0.6	0.0278
1-methylhistidine	Histidine Metabolism	0.8	0.0231
3-(4-hydroxyphenyl)lactate	Phenylalanine and Tyrosine Metabolism	0.6	0.0246
Nicotinamide ribonucleotide (NMN)	Nicotinate and Nicotinamide Metabolism	0.5	0.0247
Orotidine	Pyrimidine Metabolism, Orotate containing	0.7	0.0268
2-hydroxybutyrate/2-hydroxyisobutyrate	Methionine, Cysteine, SAM and Taurine Metabolism	0.6	0.0274
1-(1-enyl-palmitoyl)-2-arachidonoyl-GPC (P-16:0/20:4)*	Plasmalogen	0.7	0.0302
Kynurenine	Tryptophan Metabolism	0.7	0.0398
N6-acetyllysine	Lysine Metabolism	0.5	0.0372
1-(1-enyl-palmitoyl)-GPE (P-16:0)*	Lysoplasmalogen	0.7	0.0359
Adenosine 5'-monophosphate (AMP)	Purine Metabolism, Adenine containing	0.8	0.0517
1-oleoyl-GPG (18:1)*	Lysolipid	0.6	0.0525
Lactosyl-N-palmitoyl-sphingosine	Sphingolipid Metabolism	0.6	0.0604
Asparagine	Alanine and Aspartate Metabolism	0.7	0.0643
1-palmitoyl-GPG (16:0)*	Lysolipid	0.7	0.0447
Methionine sulfone	Methionine, Cysteine, SAM and Taurine Metabolism	0.8	0.0425
Glutamate, gamma-methyl ester	Glutamate Metabolism	0.7	0.0473
Nicotinamide riboside	Nicotinate and Nicotinamide Metabolism	0.6	0.0743
5-oxoproline	Glutathione Metabolism	0.9	0.0515
N-acetylphenylalanine	Phenylalanine and Tyrosine Metabolism	0.7	0.0467
3-methyl-2-oxobutyrate	Leucine, Isoleucine and Valine Metabolism	0.7	0.0522

N-acetylisoleucine	Leucine, Isoleucine and Valine Metabolism	0.7	0.0594
Methylphosphate	Purine and Pyrimidine Metabolism	0.7	0.0512
Uracil	Pyrimidine Metabolism, Uracil containing	0.7	0.0502

Supplementary Table 3

Biological Processes	KEGG pathway	p-value	Metabolites	Pathway	p-value	Fold Change
**Transcriptomic pathways and their associated metabolites**						
Proteolysis	Proteasome	0.002	Threonylphenylalanine	Dipeptide	0.04	2.3
	Ubiquitin mediated proteolysis	0.036	Valylglutamine	Dipeptide	0.02	2.1
			Glutaminylleucine	Dipeptide	0.02	2.0
			Alanylleucine	Dipeptide	0.02	1.9
			Valylglycine	Dipeptide	0.02	1.8
			Phenylalanylglycine	Dipeptide	0.04	1.8
			Phenylalanylalanine	Dipeptide	0.03	1.7
			Glycylisoleucine	Dipeptide	0.02	1.5
			Tryptophylglycine	Dipeptide	0.04	1.4
			Tyrosylglycine	Dipeptide	0.00	1.3
			Glycylvaline	Dipeptide	0.02	1.3
			Isoleucylglycine	Dipeptide	0.03	1.3
Amino Acid metabolism	Valine, leucine and isoleucine degradation	0.000	Isovaleryl CoA	Leucine, Isoleucine and Valine Metabolism	0.01	0.18
	Biosynthesis of amino acids	0.001	N-acetyltyrosine	Phenylalanine and Tyrosine Metabolism	0.02	0.45
	Lysine degradation	0.007	N6-acetyllysine	Lysine Metabolism	0.03	0.56
	Alanine, aspartate and glutamate metabolism	0.035	3-(4-hydroxyphenyl)lactate	Phenylalanine and Tyrosine Metabolism	0.02	0.63
			Glutamate, gamma-methyl ester	Glutamate Metabolism	0.02	0.72
			N-acetylleucine	Leucine, Isoleucine and Valine Metabolism	0.02	0.73
			Kynurenine	Tryptophan Metabolism	0.04	0.76
			1-methylhistidine	Histidine Metabolism	0.02	0.79
			Citrulline	Urea cycle; Arginine and Proline Metabolism	0.04	0.8
			3-methoxytyrosine	Phenylalanine and Tyrosine Metabolism	0.04	0.81
			Trans-4-hydroxyproline	Urea cycle; Arginine and Proline Metabolism	0.04	0.82
			N-acetylvaline	Leucine, Isoleucine and Valine Metabolism	0.05	0.84
			N-monomethylarginine	Urea cycle; Arginine and Proline Metabolism	0.02	0.85
			5-oxoproline	Glutathione Metabolism	0.04	0.87
			Methionine	Methionine, Cysteine, SAM and Taurine Metabolism	0.02	0.88

Gamma-glutamate dependendant amino acid transport	Gamma-glutamyl transpeptidase 1	0.001	Gamma-glutamylglutamate	Gamma-glutamyl Amino Acid	0.01	0.62
	Gamma-glutamyl transpeptidase 7	0.000	Gamma-glutamylisoleucine	Gamma-glutamyl Amino Acid	0.01	0.63
			Gamma-glutamyltyrosine	Gamma-glutamyl Amino Acid	0.01	0.66
			Gamma-glutamylthreonine	Gamma-glutamyl Amino Acid	0.03	0.70
			Gamma-glutamylphenylalanine	Gamma-glutamyl Amino Acid	0.05	0.71
			5-oxoproline	Glutathione Metabolism	0.04	0.87
