# Supplementary material for: Ribosomal mistranslation leads to silencing of the unfolded protein response and increased mitochondrial biogenesis
Source: Commun Biol. 2019 Oct 17;2:381. doi: 10.1038/s42003-019-0626-9 (PMC6797716; doi:10.1038/s42003-019-0626-9)
Supplement: Supplementary file 2 — Reporting Summary [file 42003_2019_626_MOESM2_ESM.pdf]

nature research

Corresponding author(s): Erik C. Böttger

Last updated by author(s): Sep 19, 2019

Reporting Summary

Nature Research wishes to improve the reproducibility of the work that we publish. This form provides structure for consistency and transparency in reporting. For further information on Nature Research policies, see Authors & Referees and the Editorial Policy Checklist.

Statistics

For all statistical analyses, confirm that the following items are present in the figure legend, table legend, main text, or Methods section.

n/a	Confirmed	
☐	☒	The exact sample size (n) for each experimental group/condition, given as a discrete number and unit of measurement
☐	☒	A statement on whether measurements were taken from distinct samples or whether the same sample was measured repeatedly
☐	☒	The statistical test(s) used AND whether they are one- or two-sided<br>*Only common tests should be described solely by name; describe more complex techniques in the Methods section.*
☒	☐	A description of all covariates tested
☐	☒	A description of any assumptions or corrections, such as tests of normality and adjustment for multiple comparisons
☐	☒	A full description of the statistical parameters including central tendency (e.g. means) or other basic estimates (e.g. regression coefficient) AND variation (e.g. standard deviation) or associated estimates of uncertainty (e.g. confidence intervals)
☐	☒	For null hypothesis testing, the test statistic (e.g. F, t, r) with confidence intervals, effect sizes, degrees of freedom and P value noted<br>*Give P values as exact values whenever suitable.*
☒	☐	For Bayesian analysis, information on the choice of priors and Markov chain Monte Carlo settings
☒	☐	For hierarchical and complex designs, identification of the appropriate level for tests and full reporting of outcomes
☒	☐	Estimates of effect sizes (e.g. Cohen's d, Pearson's r), indicating how they were calculated

Our web collection on statistics for biologists contains articles on many of the points above.

Software and code

Policy information about availability of computer code

Data collection	No commercial, open source or custom program code was used
Data analysis	No commercial, open source or custom program code was used for data analysis

For manuscripts utilizing custom algorithms or software that are central to the research but not yet described in published literature, software must be made available to editors/reviewers. We strongly encourage code deposition in a community repository (e.g. GitHub). See the Nature Research guidelines for submitting code & software for further information.

Data

Policy information about availability of data

All manuscripts must include a data availability statement. This statement should provide the following information, where applicable:

- Accession codes, unique identifiers, or web links for publicly available datasets
- A list of figures that have associated raw data
- A description of any restrictions on data availability

The transcriptome data are available for review under NCBI GEO accession GSE115153, token ipspauiyhjafxob.
The metabolome data are available for review under EMBL-EBI MetaboLights accession MTBLS106.

Field-specific reporting

Please select the one below that is the best fit for your research. If you are not sure, read the appropriate sections before making your selection.

☒ Life sciences ☐ Behavioural & social sciences ☐ Ecological, evolutionary & environmental sciences

Life sciences study design

All studies must disclose on these points even when the disclosure is negative.

Sample size	For every genotype at least 3 independently generated cell lines were used in every experiment.
Data exclusions	No data was excluded
Replication	Every experiment was performed at least 3 times independently
Randomization	No randomization was required
Blinding	No blinding was required

Reporting for specific materials, systems and methods

We require information from authors about some types of materials, experimental systems and methods used in many studies. Here, indicate whether each material, system or method listed is relevant to your study. If you are not sure if a list item applies to your research, read the appropriate section before selecting a response.

Materials & experimental systems

n/a	Involved in the study	
☐	☒	Antibodies
☐	☒	Eukaryotic cell lines
☒	☐	Palaeontology
☒	☐	Animals and other organisms
☒	☐	Human research participants
☒	☐	Clinical data

Methods

n/a	Involved in the study	
☒	☐	ChIP-seq
☐	☒	Flow cytometry
☒	☐	MRI-based neuroimaging

Antibodies

Antibodies used	polyclonal anti-myc (Abcam, ab9106); polyclonal anti-RPS2 (Abcam, ab154972); polyclonal anti-RPS9 (Abcam, ab117861); polyclonal anti-tubulin (Abcam, ab6046); polyclonal anti-peIF2α (Cell Signaling, 9721S); anti-eIF2α (Cell Signaling, 9722); polyclonal anti-ubiquitin (Abcam, ab7780); polyclonal anti-GAPDH (Abcam, ab9485); mouse monoclonal anti-p53 (Abcam, ab26); HRP-conjugated goat anti-rabbit (Invitrogen, G-21234) and goat anti-mouse antibodies (Invitrogen, A10551)
Validation	Validation of primary antibodies has been done by the manufacturer and can be found on the manufacturer's web site

Eukaryotic cell lines

Policy information about cell lines

Cell line source(s)	Parental cell line (HEK293) was purchased from ATCC
Authentication	No authentication was required
Mycoplasma contamination	All cell lines used in the study were tested for mycoplasma and found negative (mycoplasma-specific PCR and commercial antibody-based mycoplasma detection kit)
Commonly misidentified lines (See ICLAC register)	No commonly misidentified cell lines was used

Flow Cytometry

Plots

Confirm that:

☒ The axis labels state the marker and fluorochrome used (e.g. CD4-FITC).

☒ The axis scales are clearly visible. Include numbers along axes only for bottom left plot of group (a 'group' is an analysis of identical markers).

☒ All plots are contour plots with outliers or pseudocolor plots.

☒ A numerical value for number of cells or percentage (with statistics) is provided.

Methodology

Sample preparation	Following experiment-specific treatment, the cells were detached from surface using Accutase treatment, suspended in PBS buffer and used for FACS analysis (alive)
Instrument	FACS LSR Fortessa, BD Biosciences
Software	FACSDIVA version 8.0 was used for data collection; FlowJo version 10.0 was used for analysis
Cell population abundance	Only homogeneous cell populations were used in the study
Gating strategy	Example of gating strategy could be provided in a supplementary information file upon request

☐ Tick this box to confirm that a figure exemplifying the gating strategy is provided in the Supplementary Information.
