# Supplementary material for: Ribosomal mistranslation leads to silencing of the unfolded protein response and increased mitochondrial biogenesis
Source: Commun Biol. 2019 Oct 17;2:381. doi: 10.1038/s42003-019-0626-9 (PMC6797716; doi:10.1038/s42003-019-0626-9)
Supplement: Supplementary file 3 — Description of Additional Supplementary Items [file 42003_2019_626_MOESM3_ESM.docx]

**Description of additional supplementary files**

**File name:** Supplementary Data 1

**Description:** List of genes sorted by pathways.

**File name:** Supplementary Data 2

**Description:** Source data underlying the graphs presented in the main figures.
